# Analysis of bioactive compounds in cinnamon leaves and preparation of nanoemulsion and byproducts for improving Parkinson’s disease in rats

**DOI:** 10.3389/fnut.2023.1229192

**Published:** 2023-08-02

**Authors:** Yi Chun Wang, Vinchi Wang, Bing Huei Chen

**Affiliations:** ^1^Department of Food Science, Fu Jen Catholic University, New Taipei City, Taiwan; ^2^School of Medicine, Fu Jen Catholic University, New Taipei City, Taiwan; ^3^Department of Nutrition, China Medical University, Taichung, Taiwan

**Keywords:** cinnamon leaves, hydrosol, nanoemulsion, UPLC-MS/MS, Parkinson’s disease in rats

## Abstract

**Introduction:**

*Cinnamomum osmophloeum* Kanehira (C. osmophloeum), a broad-leaved tree species of Taiwan, contains phenolic acids, flavonoids, and phenylpropanoids such as cinnamaldehyde and cinnamic acid in leaves. Many reports have shown that the cinnamon leaf extract possesses anti-inflammatory, hypoglycemic, hypolipidemic and neuroprotective functions. This study aims to analyze bioactive compounds in C. osmophloeum (cinnamon leaves) by UPLC-MS/MS and prepare hydrosol, cinnamon leaf extract and cinnamon leaf nanoemulsion for comparison in improving Parkinson’s disease (PD) in rats.

**Methods:**

After extraction and determination of total phenolic and total flavonoid contents, cinnamaldehyde and the other bioactive compounds were analyzed in cinnamon leaves and hydrosol by UPLC-MS/MS. Cinnamon leaf nanoemulsion was prepared by mixing a suitable proportion of cinnamon leaf extract, soybean oil, lecithin, Tween 80 and deionized water, followed by characterization of particle size and polydispersity index by dynamic light scattering analyzer, particle size and shape by transmission electron microscope, encapsulation efficiency, as well as storage and heating stability. Fifty-six male Sprague-Dawley rats aged 8 weeks were divided into seven groups with group 1 as control (sunflower oil) and group 2 as induction (2 mg/kg bw rotenone in sunflower oil plus 10 mL/kg bw saline), while the other groups including rotenone injection (2 mg/kg bw) followed by high-dose of 60 mg/kg bw (group 3) or low-dose of 20 mg/kg bw (group 4) for tube feeding of cinnamon leaf extract or cinnamon leaf nanoemulsion at the same doses (groups 5 and 6) every day for 5 weeks as well as group 7 with rotenone plus hydrosol containing 0.5 g cinnamon leaf powder at a dose of 10 mL/kg bw. Biochemical analysis of brain tissue (striatum and midbrain) was done to determine dopamine, α-synuclein, tyrosine hydroxylase, superoxide dismutase, catalase, glutathione peroxidase and malondialdehyde contents by using commercial kits, while catalepsy performed by bar test.

**Results and discussion:**

An extraction solvent of 80% ethanol was found to be the most optimal with a high yield of 15 bioactive compounds being obtained following UPLC analysis. A triple quadrupole tandem mass spectrometer with electrospray ionization mode was used for identification and quantitation, with cinnamaldehyde present at the highest amount (17985.2 µg/g). The cinnamon leaf nanoemulsion was successfully prepared with the mean particle size, zeta potential, polydispersity index and encapsulation efficiency being 30.1 nm, -43.1 mV, 0.149 and 91.6%, respectively. A high stability of cinnamon leaf nanoemulsion was shown over a 90-day storage period at 4 and heating at 100 for 2 h. Animal experiments revealed that the treatments of cinnamon leaf extract, nanoemulsion and hydrosol increased the dopamine contents from 17.08% to 49.39% and tyrosine hydroxylase levels from 17.07% to 25.59%, while reduced the α-synuclein levels from 17.56% to 15.95% in the striatum of rats. Additionally, in the midbrain of rats, an elevation of activities of superoxide dismutase (6.69-16.82%), catalase (8.56-16.94%), and glutathione peroxidase (2.09-16.94%) was shown, while the malondialdehyde content declined by 15.47-22.47%. Comparatively, the high-dose nanoemulsion exerted the most pronounced effect in improving PD in rats and may be a promising candidate for the development of health food or botanic drug.

## 1. Introduction

*Cinnamomum osmophloeum* (*C. osmophloeum*), a broad-leaved tree species endemic to Taiwan, belongs to the *Lauraceae* family and contains many biologically active compounds. Studies have shown that the cinnamon leaves contained a variety of bioactive compounds such as cis-cinnamaldehyde, trans-cinnamaldehyde, isobornyl acetate, eugenol, cinnamyl acetate, phenolic acids and flavonoids (1), while the essential oil extracted from cinnamon leaves as identified by GC/MS was found to contain monoterpenoids, sesquiterpenoids, alcohols, phenols, aldehydes, ketones, esters, acids, and other compounds (2). Most importantly, cinnamon leaves have been shown to possess vital biological activities such as anti-cancer, anti-diabetes, neuroprotection, and cardiovascular protection (3–6).

Nanotechnology is defined as the design of characteristic products for application in the fields of food, drug, cosmetics and medicine by controlling the shape and size to 1–100 nm (7). When the material is at the nanometer level, its surface area and the number of surface atoms will increase greatly which in turn modify the original physical and chemical properties, thereby achieving higher physiological activity and bioavailability (8). Of the various nanosystems, nanoemulsion is the most common one frequently used. Theoretically, nanoemulsion can form a transparent, stable and uniform dispersion system with a size from 10–100 nm by using surfactants in two incompatible liquids, the oil phase and the water phase (9). As the mean size of human cells is 10–100 μm, the employment of nanoemulsion can be expected to facilitate delivery of drugs through the blood–brain barrier (BBB) (10). Furthermore, many bioactive compounds suffer the drawback of poor water solubility and stability, as well as, bioavailability *in vivo*, which limit their applications in food and drug industries. Compared to traditional emulsion, nanoemulsion possesses the capability of encapsulating bioactive compounds to improve the stability and bioavailability *in vivo* (11, 12). In addition, the release of bioactive compounds or drugs can be controlled in a slow and prolonged pattern, thus both the frequency and dosage can be reduced throughout the course of treatment, while the possible side effect can be minimized as well (13).

Parkinson’s disease (PD), a neurodegenerative disease, is becoming more common worldwide due to a rise in aging population. The World Health Organization statistics show that about 18 million people are expected to suffer from PD in 2030 and the number continues to rise by multiple growths every year, thereby creating a huge burden for health care in many countries (14). According to a health insurance statistics report published by Taiwan Food and Drug Administration, the total number of PD patients was 77,428 in 2021 and continued to grow every year, which can be closely associated with the Taiwan’s aging populations (15). Furthermore, in Taiwan, the incidence of PD was shown to rise with age with men being more prone to PD than women. Also, those who are not in the labor force or with low income were prone to PD due to an increased adjusted-incidence-rate-ratio of 1.50–1.56 and adjusted-prevalence-rate-ratio of 1.66–1.71 (16).

PD, a movement disorder, is characterized by the loss of dopaminergic neurons in the substantia nigra of the midbrain, which can be attributed to the accumulation of α-synuclein in intracellular deposits known as Lewy bodies and Lewy neurites (17). The symptoms associated with inadequate secretion of dopamine will appear such as the elevation of oxidative stress, possibly caused by the overproduction of free radicals *in vivo* reacting with lipids in the brain cell membrane to generate lipid hydroperoxides and degradation products, leading to cell membrane damage and neuron death (18). Additionally, a high iron and low glutathione content was found in the substantia nigra of PD patients, and an excessive amount of iron has been shown to accelerate production of lipid hydroperoxides (19). Therefore, the insufficient intake of antioxidants may also lead to the death of dopamine neurons. Over the years, many natural antioxidants such as phenolic acids and flavonoids present in plants are believed to enhance cognitive performance and maintain brain health (20). The ability to scavenge free radicals and the presence of major bioactive compounds such as cinnamaldehyde can make cinnamon plant a potential material for treatment of PD (21).

As mentioned above, cinnamon plant is rich in cinnamaldehyde and a significant amount of phenolic acids and flavonoids is shown, but due to poor water solubility of cinnamaldehyde, the possible treatment of PD by cinnamon leaves may be invalid. The aim of this study was to extract cinnamaldehyde and the other bioactive compounds from cinnamon leaves, followed by identification and quantitation by UPLC-MS/MS. Also, the cinnamon leaf extract, cinnamon leaf nanoemulsion and hydrosol (a distilled product from cinnamon leaves) were prepared to compare their efficiency in improving PD in rats.

## 2. Materials and methods

### 2.1. Analysis of cinnamaldehyde and the other bioactive compounds in cinnamon leaves and hydrosol

#### 2.1.1. Processing of cinnamon leaves

Fu-Tai Co. (Taipei, Taiwan) provided the cinnamon leaves for this study which was harvested in November from Pinlin district and Taitung County, Taiwan. A total of 1 kg cinnamon leaves was transported to our lab, cleaned, oven-dried at 60°C for 2 h and ground into powder (550 g) for subsequent experiments. The moisture contents in fresh and oven-dried leaves were 45.1 and 5.8%, respectively.

#### 2.1.2. Determination of total phenolic content in cinnamon leaves

The TPC in cinnamon leaves was determined by Folin–Ciocalteu’s phenol method as reported by Abeysekera et al. (22) and Kao et al. (23) and expressed as gallic acid equivalent. Initially a total of 5 gallic acid standard (in ethanol) concentrations including 50, 100, 200, 300, and 400 μg/mL were prepared, after which a portion (50 μL each) was collected and mixed with 200 μL of Folin’s phenol reagent. Following mixing evenly in the dark for 5 min, 1 mL of sodium carbonate solution (15%) was added, followed by mixing well, keeping at room temperature in the dark for 1 h, measuring the absorbance at 750 nm, and a standard curve was prepared by plotting concentration against absorbance to obtain the linear regression equation and the coefficient of determination. Next, a 50 μL of sample extract was collected and the same procedure was followed for the absorbance measurement at 750 nm for calculation of TPC using the linear regression equation of the gallic acid standard curve.

#### 2.1.3. Determination of total flavonoid content in cinnamon leaves

The TFC in cinnamon leaves was determined based on two methods reported by Abeysekera et al. (22) and Kao et al. (23) and expressed as quercetin equivalent. Initially a total of 6 quercetin standard (in ethanol) concentrations including 5, 10, 25, 50, 100, and 200 μg/mL were prepared, after which a portion (200 μL each) was collected and mixed with 30 μL of sodium nitrite solution (5%). Then this mixture was left at room temperature for 5 min, followed by adding 60 μL of aluminum chloride solution (10%), standing at room temperature for 5 min, adding 300 μL of sodium hydroxide solution (1 M) and 200 μL of chloroform, mixing evenly, centrifuging, collecting the supernatant and measuring the absorbance at 510 nm. Then the quercetin standard curve was prepared by plotting concentration against absorbance to obtain the linear regression equation and coefficient of determination. Next, a 200 μL of sample extract was collected and the same procedure was followed for the absorbance measurement at 510 nm for calculation of TFC using the linear regression equation of the quercetin standard curve.

#### 2.1.4. Extraction of cinnamaldehyde and the other bioactive compounds in cinnamon leaves and hydrosol

Cinnamon leaf nanoemulsion is mainly composed of cinnamon leaf extract, Tween 80, soybean oil, lecithin and deionized water. Of the various bioactive compounds, cinnamaldehyde is the dominant one in both cinnamon leaf extract and cinnamon leaf nanoemulsion. However, the prepared nanoemulsion was used to encapsulate cinnamaldehyde and the other bioactive compounds in the extract for elevation of their stability and bioavailability *in vivo*. Furthermore, the cinnamaldehyde content remains the same in both nanoemulsion and extract for the animal study for comparison of the efficiency in improving PD.

A method reported by Waty and Suryanto (24) was modified and used for the extraction of bioactive compounds including cinnamaldehyde, phenolic acids, and flavonoids from cinnamon leaves. To 1 g of cinnamon leaf powder, 30 mL of 80% methanol or 80% ethanol solution was added and the mixture was sonicated at 60°C for 2 h, and then centrifuged at 4000 rpm at 25°C for 20 min. This procedure was repeated 3 times and the combined supernatants were collected for filtration using a filter paper and evaporation of solvent under nitrogen. The residue was then dissolved in 10 mL of 80% methanol or 80% ethanol and analyzed for individual bioactive compounds by UPLC-MS/MS. In a previous study dealing with analysis of bioactive compounds in cinnamon leaves grown in Pinlin district, New Taipei City, Taiwan, a much higher amount of cinnamaldehyde and the other bioactive compounds was shown with 80% ethanol as the extraction solvent compared with 30% ethanol as the extraction solvent (4). Thus, in this study we only compared the effect of 80% ethanol and 80% methanol on the contents of cinnamaldehyde and the other bioactive compounds in cinnamon leaves grown in Taitung County, Taiwan. Whereas, the hydrosol containing 97% of water was directly analyzed by UPLC-MS/MS without solvent extraction.

#### 2.1.5. UPLC-MS/MS analysis

Using a Luna Omega C18 100 Å LC column (100 × 2.1 mm ID,1.6 μm particle size) with 0.025% acetic acid in water (A) and 0.025% acetic acid in methanol (B) as mobile phase, flow rate at 0.3 mL/min, column temperature at 30°C, the elution was carried out in a gradient mode with 83% A and 17% B initially, raised to 20% B in one min, 40% B in 5 min, 55% B in 10 min, 99% B in 14 min, and finally returned to the initial ratio. A total of 15 components were separated within 14 min, including 5-O-caffeoylquinic acid, caffeic acid, p-coumaric acid, coumarin, benzoic acid, rutin, quercetin 3-O-galactoside, quercetin 3-O-glucoside, quercetin, cinnamyl alcohol, cinnamaldehyde, kaempferol, kaempferol-3-β-D-glucopyranoside, trans-cinnamic acid and eugenol. But the peaks of quercetin-3-O-galactoside and quercetin-3-O-glucoside overlapped.

The MS/MS detection by multiple reaction monitoring (MRM) mode was done in both ESI positive and negative ions as reported by Huang and Chen (4). Identification of cinnamaldehyde and the other bioactive components in cinnamon leaves and hydrosol was performed by comparing the retention time and mass spectra of standards with those of unknown peaks on the UPLC chromatogram.

#### 2.1.6. Quantitation of bioactive compounds in cinnamon leaves and hydrosol

All the standards including 5-O-caffeoylquinic acid, caffeic acid, p-coumaric acid, coumarin, benzoic acid, rutin, quercetin 3-O-galactoside, quercetin 3-O-glucoside, quercetin, cinnamyl alcohol, cinnamaldehyde, kaempferol, kaempferol-3-β-D-glucopyranoside, trans-cinnamic acid and eugenol were each dissolved in 80% ethanol and 8 concentrations including 500, 400, 300, 200, 100, 50, 20, and 10 ng/mL for each standard were prepared. After UPLC-MS/MS analysis, the calibration curves were obtained by plotting concentration against peak area and the linear regression equations used for calculation of the content of each bioactive compound were *y* = 95.239*x*–1811.4 for 5-O-caffeoylquinic acid, *y* = 575.78*x* + 663.16 for caffeic acid, *y* = 172.24*x* + 500.63 for p-coumaric acid, *y* = 868.53*x*–12,024 for coumarin, *y* = 96*x* + 1360.7 for benzoic acid, *y* = 349.3*x* + 593.46 for rutin, *y* = 447.31*x*–936.38 for quercetin 3-O-galactoside, *y* = 447.31*x*–936.38 for quercetin 3-O-glucoside, *y* = 21.489*x*–242.09 for quercetin, *y* = 135.37*x* + 3793.5 for cinnamyl alcohol, *y* = 120.98*x*–576.95 for cinnamaldehyde, *y* = 1413.6*x* + 2789.1 for kaempferol, *y* = 447.87*x*–2,624 for kaempferol-3-β-D-glucopyranoside, *y* = 200.98*x*–137.52 for trans-cinnamic acid and *y* = 9.5249*x* + 61.521 for eugenol.

### 2.2. Preparation of cinnamon leaf extract, nanoemulsion, and hydrosol

The cinnamon leaf extract containing 5,000 ppm cinnamaldehyde extract (based on UPLC analysis) was prepared by mixing 100 g of dried cinnamon leaves with 500 mL of 80% ethanol and stirring for 1 h, after which a portion (20 mL) was collected for preparation of cinnamon leaf nanoemulsion. To prepare 10 mL of cinnamon leaf nanoemulsion containing 10,000 ppm of cinnamaldehyde, 0.1 g (1%) of soybean oil, 0.2 g (2%) of lecithin and 0.6 g (6%) of Tween 80 were mixed and dissolved in 99% ethanol, followed by mixing thoroughly in a round-bottomed flask, adding 20 mL of the cinnamon leaf extract, mixing thoroughly, evaporating to dryness under vacuum to form a thin film, adding 9.1 g of deionized water (91%), and ultrasonicating for 30 min. Hydrosol (30 L) was the aqueous portion obtained through separation from essential oil by mixing 8 kg of fresh cinnamon leaves with 50 L of pure water in a distiller for steam distillation at 100°C for 2 h. Due to the presence of a large proportion of water (97%) and lower amount of cinnamaldehyde and the other bioactive compounds in hydrosol, 0.5 g of cinnamon leaf powder was dissolved in 10 mL of hydrosol to elevate the content of cinnamaldehyde and the other compounds. Specifically, the cinnamaldehyde content in hydrosol was increased to 17 mg/kg to attain a dose of 10 mL/kg bw for tube feeding in rats.

### 2.3. Characterization of cinnamon leaf nanoemulsion

#### 2.3.1. Particle size and shape of cinnamon leaf nanoemulsion

The prepared nanoemulsion was characterized for both particle size and polydispersity index (PDI) in a dynamic light scattering (DLS) analyzer by diluting 50 times with 25 mM of KH_2_PO_4_ buffer (pH 5.5), filtering through a 0.22 μm membrane filter and pouring into a polystyrene colorimetric tube for analysis. A portion (100 μL) of this nanoemulsion was diluted 200 times with deionized water for determination of zeta potential at 25°C. In addition, by using a transmission electron microscope (TEM), both the particle size and shape of the nanoemulsion was determined by diluting 50 times with deionized water and 20 μL was collected and dropped on a carbon-coated copper grid. After 90 s, the excessive sample was removed with a filter paper for negative staining with 20 μL of phosphotungstic acid (2%) for 30 s, followed by removing excessive stain with a filter paper and drying overnight in an oven.

#### 2.3.2. Encapsulation efficiency of cinnamaldehyde

Initially, free cinnamaldehyde was extracted from the nanoemulsion by mixing 400 μL of n-hexane and 100 μL of nanoemulsion for dissolving cinnamaldehyde into the n-hexane phase (upper layer). In addition, 100 μL of nanoemulsion was mixed with 400 μL of ethanol and the mixture was ultrasonicated for 2 h to release total cinnamaldehyde for analysis by HPLC with UV detection at 280 nm. The encapsulation efficiency of cinnamaldehyde was then calculated using the formula shown below:

Encapsulation efficiency of cinnamaldehyde.


$$=\frac{\;\mathrm{total amount of cinnamaldehyde}-\mathrm{free cinnamaldehyde}}{\mathrm{total amount of cinnamaldehyde}}\times 100$$

#### 2.3.3. Stability of cinnamon leaf nanoemulsion

The storage stability was determined by pouring a 1 mL nanoemulsion into an eppendorf tube in triplicate and storing for a total of 18 tubes at 4°C for 3 mo for subsequent analysis of mean particle size, polydispersity index and zeta potential every week, while the heating stability was conducted by collecting a 0.2 mL nanoemulsion in an eppendorf tube and heating in a water bath separately at 40, 70, and 100°C for 0.5, 1, 1.5, and 2 h in triplicate for a total of 36 tubes, and analyzing the above 3 parameters for each heating time length.

### 2.4. Animal study

Fifty-six male Sprague–Dawley rats, aged 6 weeks, was obtained from Taiwan BioLASCO Co (Taipei, Taiwan), and these rats were individually housed in ventilated cages at Fu Jen University Animal Center maintained at a temperature of 21 ± 2°C and relative humidity of 55 ± 10% in a 12 h dark/light cycle. The ethical approval to conduct this animal study was previously obtained from the Fu Jen University Animal Care and Use Committee (permission no. A11053) and all the procedures adhered to approved guidelines. Throughout the study, the rats were provided with a standard laboratory rodent diet (LabDiet Co, St Louis, MO, United States) and uninterrupted access to water. Weekly measurements of body weight and water intake was recorded over a period of 5 weeks. After a 2 week acclimatization period, the rats reached 8 weeks of age and were ready for conducting experiments.

#### 2.4.1. Experiment design

The rats were then randomly divided into seven different groups with 8 rats each: Group 1 (control group): subcutaneous (SC) injection with sunflower oil at a dose of 1 mL/kg body weight (bw) every day followed by tube feeding 30 min later with normal saline at a dose of 10 mL/kg bw as a vehicle for a period of 5 weeks; Group 2 (rotenone-induced group): SC injection with rotenone at a dose of 2 mg/kg bw (dissolved in sunflower oil at 2 mg/mL) every day followed by tube feeding 30 min later with normal saline (10 mL/kg bw) for 5 weeks successively; Group 3: SC injection with rotenone (2 mg/kg bw) every day followed by tube feeding 30 min later with HDCE (high-dose cinnamon leaf extract) at 60 mg/kg bw for 5 weeks successively; Group 4: SC injection with rotenone (2 mg/kg bw) every day followed by tube feeding 30 min later with LDCE (low-dose cinnamon leaf extract) at 20 mg/kg bw for 5 weeks; Group 5: SC injection with rotenone (2 mg/kg bw) every day followed by tube feeding 30 min later with HDCN (high-dose cinnamon leaf nanoemulsion) at 60 mg/kg bw for 5 weeks; Group 6: SC injection with rotenone (2 mg/kg bw) every day followed by tube feeding 30 min later with LDCN (low-dose cinnamon leaf nanoemulsion) at 20 mg/kg bw for 5 weeks; Group 7: SC injection with rotenone (2 mg/kg bw) every day followed by tube feeding 30 min later with cinnamon leaf powder dissolved in hydrosol (0.5 g/10 mL) at a dose of 10 mL/kg bw for 5 weeks.

#### 2.4.2. Catalepsy test

Catalepsy test was assessed using the bar test, in which the rats were positioned in a half-rearing stance with their front paws resting on a horizontal bar located 9 cm above and parallel to the bottom surface. The rats were then carefully observed to measure the duration until one paw was lifted from the bar. After the maximum cut-off time of 180 s for observation, the rats were subjected to carbon dioxide asphyxiation before sacrifice and then both striatum and midbrain were collected separately and weighed, followed by adding homogenate for extraction with a homogenizer. The extracts were then stored at −80°C for subsequent analysis.

#### 2.4.3. Biochemical analysis in brain tissue

Dopamine content was analyzed with a commercial kit (EU0392, Fine Biotech Co., Wuhan, China). In brief, the antibody-coated 96-well plate was washed with wash buffer twice, followed by adding 50 μL of samples or standards of each concentration (0, 1.562, 3.125, 6.25, 12.5, 25, 50, 100 ng/mL), 50 μL of antibody (biotin-labeled antibody working solution), mixing and reacting at 37°C for 45 min, washing with wash buffer solution 3 times, adding 100 μL of horseradish peroxidase polymer (HRP-streptavidin conjugate), mixing at 37°C for 30 min, washing again with wash buffer solution 5 times, adding 90 μL of TMB (3,3′,5,5′-tetramethylbenzidine) substrate, reacting at 37°C for 15 min in the dark, adding 50 μL of stop solution, and the absorbance was measured at 450 nm immediately after mixing. Then the dopamine content of each sample was calculated based on the standard curve.

The 𝛼-synuclein content was analyzed with a commercially available kit (ER0921, Fine Biotech Co., Wuhan, China). Briefly, the antibody-coated 96-well plate was washed with wash buffer solution twice, and then 100 μL of samples or standards of each concentration (0, 15.625, 31.25, 62.5, 125, 250, 500, 1,000 pg./mL) were added and reacted at 37°C for 90 min. Each solution was washed twice with wash buffer solution and mixed with 100 μL of antibody (biotin-labeled antibody working solution) for reaction at 37°C for 60 min, followed by washing 3 times with wash buffer solution, adding 100 μL of horseradish peroxidase polymer (HRP-streptavidin conjugate), reacting at 37°C for 30 min, washing 5 times with wash buffer solution, adding 90 μL of TMB as substrate, reacting at 37°C for 15 min in the dark, adding 50 μL of the stop reagent, and measuring the absorbance at 450 nm immediately after mixing. The content of 𝛼-synuclein in each sample was then calculated based on the standard curve.

Tyrosine hydroxylase (TH) content was also analyzed with a commercial kit (ER0534, Fine Biotech Co., Wuhan, China). Briefly, the antibody-coated 96-well plate was washed twice with wash buffer solution, and 100 μL of samples or standards of each concentration (0, 0.156, 0.312, 0.625, 1.25, 2.5, 5, 10 ng/mL) were added for reaction at 37°C for 90 min, followed by washing twice with wash buffer solution twice, adding 100 μL of antibody (biotin-labeled antibody working solution), reacting at 37°C for 60 min, washing 3 times with wash buffer solution, adding 100 μL of horseradish peroxidase polymer (HRP-streptavidin conjugate), reacting at 37°C for 30 min, washing 5 times with wash buffer solution, adding 90 μL of TMB as substrate, reacting at 37°C for 15 min in the dark, adding 50 μL of the stop reagent, and measuring the absorbance at 450 nm immediately after mixing. Then, the TH content in each sample was calculated based on the standard curve.

Superoxide dismutase (SOD) activity was assayed with a commercial kit (706,002, Cayman Chemical Co., Ann Arbor, MI, United States). One unit of SOD is defined as the amount of enzyme required for 50% superoxide radical dismutation. In brief, the bovine erythrocyte SOD (Cu/Zn) standard including 0.005, 0.010, 0.020, 0.030, 0.040, and 0.050 U/mL were prepared separately. Then a 10 μL of sample or each concentration from standard was collected and added to a 96-well plate, and then 200 μL of the radical detector was added. After mixing thoroughly, 20 μL of xanthine oxidase was added for the initial reaction to proceed, followed by shaking at room temperature for 30 min, and the absorbance at 450 nm was measured. Then the SOD activity of each sample was measured based on the standard curve.

Catalase (CAT) activity was assayed with a commercial kit (707,002, Cayman Chemical Company, Ann Arbor, MI, United States). Initially, a total of 6 concentrations of the formaldehyde standard including 5, 15, 30, 45, 60, and 75 μM were prepared separately. Then a 20 μL sample or standard was added to a 96-well plate, followed by adding 100 μL of buffer and 30 μL of methanol, mixing well and then adding 20 μL of hydrogen peroxide to start the reaction, shaking at room temperature for 20 min, adding 30 μL of potassium hydroxide to stop the reaction, adding 30 μL of catalase purpald, shaking for 10 min at room temperature, and finally adding 10 μL of potassium periodate. After 5 min, the absorbance at 540 nm was measured. Then the CAT activity of each sample was calculated based on the standard curve.

Glutathione peroxidase (GSH-Px) activity was assayed with a commercially available kit (703,102, Cayman Chemical Co., Ann Arbor, MI, United States). In brief, a 20 μL sample or GSH-Px standard was added to a 96-well plate, followed by adding 50 μL of buffer, 50 μL of co-substrate mixture and 50 μL of NADPH, mixing well and then adding 20 μL of cumene hydroperoxide to start the reaction. After shaking at room temperature for a few seconds, the absorbance at 340 nm was measured once every minute for 5 times for each concentration. Then the values at two time points were used to calculate the GSH-Px activity of each sample.

The malondialdehyde (MDA) content was analyzed with a commercial kit (700,870, Cayman Chemical Co., Ann Arbor, MI, United States). Briefly, a total of 7 MDA standard concentrations including 0.625, 1.25, 2.5, 5, 10, 25, and 50 μM were prepared separately. Then a 100 μL sample or standard was collected and mixed with 100 μL of the trichloroacetic acid reagent and 800 μL of the coloring reagent, after which the mixture was boiled in a water bath (100°C) for 1 h, followed by cooling on ice for 10 min and centrifuging at 1600 g at 4°C for 10 min. The supernatant (200 μL) was collected and added to a 96-well plate, and the absorbance at 535 nm was measured. Then the MDA content in each sample was calculated based on the standard curve.

### 2.5. Statistical analysis

Statistical Analysis System (SAS) with a statistical software package (25) was used for analysis of variance (ANOVA), and Duncan’s multiple range test to analyze the mean data for significant difference (*p* < 0.05).

## 3. Results and discussion

### 3.1. TPC and TFC in cinnamon leaves

The effect of 80% methanol or 80% ethanol on both TPC and TFC in cinnamon leaves is presented in Table 1. Compared to 80% methanol (17.52 mg/g), the use of 80% ethanol as the extraction solvent could result in a significantly higher TPC (22.27 mg/g). A similar result was found for TFC with the level being 25.81 mg/g for 80% ethanol and 15.32 mg/g for 80% methanol. Apparently, a higher yield of TPC and TFC in cinnamon leaves was obtained with 80% ethanol as the extraction solvent. In a previous study a higher content of TPC (44.57 mg/g) and lower content of TFC (12.00 mg/g) in cinnamon leaves from Ceylon was shown with 95% ethanol, while for cinnamon bark, the TPC and TFC were 33.43 and 3.07 mg/g, respectively (26). Likewise, the TPC in *Cinnamomum cassia* bark was reported to be higher with 80% ethanol than that with 50% or 100% ethanol (27). As expected, the Ceylon cinnamon leaves showed a higher TPC, but a lower TFC than the Taiwan cinnamon leaves used in this study, which may be caused by the difference in cinnamon species, growth location, harvesting time or variety of extraction solvent.

**Table 1:** Effects of different solvents on total phenolic content (TPC) and total flavonoid content (TFC) in cinnamon leaves.

	80% EtOH^a^^,^^b^	80% MeOH
TPC^c^	22.27 ± 0.72^a^	17.52 ± 0.54^b^
TFC^d^	25.81 ± 0.15^a^	15.32 ± 0.54^b^

a Data are presented as mean ± standard deviation of triplicate determinations.

b Data with different small letters (a)–(b) in the same row are significantly different at *p* < 0.05.

c Data expressed as mg/g of gallic acid equivalent (GAE).

d Data expressed as mg/g of quercetin equivalent (QE).

### 3.2. Analysis of bioactive compounds in cinnamon leaves by UPLC-MS/MS

**Figure 1:**
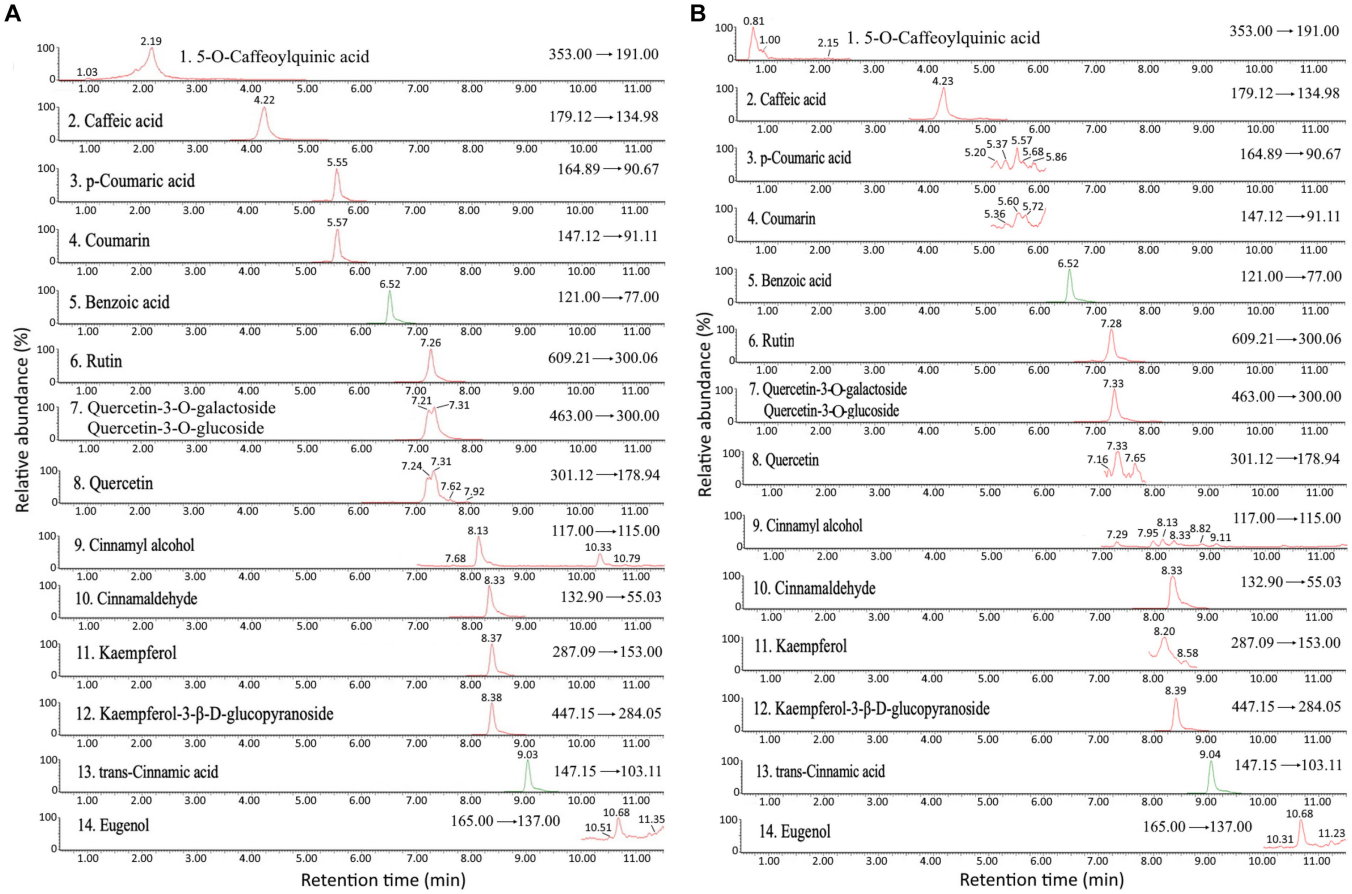
Ultra performance liquid chromatography-mass spectrometry/mass spectrometry (UPLC-MS/MS) chromatogram of standards **(A)** and cinnamon leaf extract **(B)** detected by multiple reaction monitoring (MRM) mode.

A total of 15 bioactive compounds in cinnamon leaves including 5-O-caffeoylquinic acid, caffeic acid, p-coumaric acid, coumarin, benzoic acid, rutin, quercetin-3-O-galactoside, quercetin3-O-glucoside, quercetin, cinnamyl alcohol, cinnamaldehyde, kaempferol, kaempferol-3-β-D-glucopyranoside, trans-cinnamic acid and eugenol were separated and identified with the retention time ranging from 2.15–10.68 min (Table 2; Figure 1). Identification of each bioactive compound was based on comparison of mass spectral data with that reported by Huang and Chen (4).

**Table 2:** Identification and quantitation data of cinnamaldehyde and the other bioactive compounds in cinnamon leaf extract and hydrosol by UPLC-MS/MS.

Peak no.	Compound^a^	Retention Time (min)	MS/MS (m/z)			Content (μg/g)^c^		
			Precursor ion	Product ion	Reported^b^	80% Ethanol	80% Methanol	Hydrosol^e^
1	5-O-Caffeoylquinic acid	2.15	353	179	353, 179	0.3 ± 0.11	ND^d^	ND
2	Caffeic acid	4.23	179	134	179, 134	2.0 ± 0.06	7.6 ± 0.07	ND
3	p-Coumaric acid	5.57	164	90	164, 90	3.9 ± 0.69	ND	ND
4	Coumarin	5.60	147	91	147, 91	2.0 ± 0.10	ND	ND
5	Benzoic acid	6.52	121	77	121, 77	56.4 ± 0.42	22.8 ± 0.51	3.57 ± 1.51
6	Rutin	7.28	609	300	609, 300	4.0 ± 0.08	1.7 ± 0.04	ND
7	Quercetin-3-O-galactoside Quercetin-3-O-glucoside	7.33	463	300	463, 300	4.6 ± 0.15	3.7 ± 0.04	ND
8	Quercetin	7.33	301	151	301, 151	16.6 ± 1.52	3.5 ± 0.16	ND
9	Cinnamyl alcohol	8.13	117	115	117, 115	76.8 ± 4.41	71.2 ± 2.88	3.36 ± 0.42
10	Cinnamaldehyde	8.33	132	55	132, 55	17985.2 ± 330.33	9963.3 ± 150.44	1099.60 ± 82.55
11	Kaempferol	8.20	287	153	287, 153	4.1 ± 0.48	0.4 ± 0.07	ND
12	Kaempferol-3-β-D-glucopyranoside	8.39	447	284	447, 284	16.6 ± 0.30	16.1 ± 0.22	ND
13	trans-Cinnamic acid	9.04	147	103	147, 103	387.4 ± 5.61	185.1 ± 3.96	1.62 ± 0.07
14	Eugenol	10.68	165	137	165, 137	183.4 ± 1.61	72.2 ± 6.75	12.06 ± 0.18

a Peaks were positively identified based on comparison of retention time and mass spectra of standards with unknown peaks.

b Reported MS/MS data were based on a reference by Huang and Chen (4).

c Data are presented as mean ± standard deviation of triplicate determinations.

d Not detected.

e Directly analyzed by UPLC-MS/MS without solvent extraction.

The quantitative data of various bioactive compounds extracted from cinnamon leaves with 80% ethanol is shown in Table 2, with the contents of 5-O-caffeoylquinic acid, caffeic acid, p-coumaric acid, coumarin, benzoic acid, rutin, quercetin-3-O-galactoside plus quercetin-3-O-glucoside, quercetin, cinnamyl alcohol, cinnamaldehyde, kaempferol, kaempferol-3-β-D-glucopyranoside, trans-cinnamic acid and eugenol being 0.3, 2.0, 3.9, 2.0, 56.4, 4.0, 4.6, 16.6, 76.8, 17985.2, 4.1, 16.6, 387.4, and 183.4 μg/g, respectively. Table 2 also presents the contents of individual bioactive compounds extracted from cinnamon leaves and hydrosol with 80% methanol. Like TPC and TFC, 80% ethanol could yield a much higher content of bioactive compounds such as cinnamaldehyde (17985.2 μg/g) and cinnamyl alcohol (76.8 μg/g) compared to 80% methanol. Thus, 80% ethanol was chosen as an appropriate extraction solvent for all further experiments in this study. An analogous outcome was also reported by Kim et al. (28), showing that compared to pure water (100%), 30, 50, and 100% ethanol, 80% ethanol was the most effective in extracting cinnamaldehyde (11521.08 μg/g) from cinnamon powder. However, a study comparing the effect of different extraction methods on the yield of phenolic compounds from cinnamon bark in China reported a high content of trans-cinnamic acid (103 μg/g) with 60% ethanol as the extraction solvent, temperature at 50°C for 90 min and sample to solvent ratio at 1:20 (w/v) (29).

The contents of individual bioactive compound in hydrosol is also shown in Table 2, with cinnamaldehyde being present in the largest amount (1099.60 μg/g), followed by eugenol (12.06 μg/g), benzoic acid (3.57 μg/g), and cinnamyl alcohol (3.36 μg/g). However, the hydrosol collected from Nantou County, Taiwan and analyzed by GC–MS was shown to contain cinnamaldehyde, benzaldehyde and cinnamyl acetate with the levels accounting for 87.7, 7.0, and 5.3%, respectively (30). In another study Shahpar et al. (31) analyzed chemical components of hydrosol from Iran market and reported that cinnamaldehyde was the most abundant one accounting for 63.04–91.61%. The difference in cinnamaldehyde content in hydrosol can be dependent upon cinnamon leaf species and distillation condition.

In the literature reports many studies have shown that cinnamon tree barks and leaves were effective in improving chronic diseases such as neurodegeneration, diabetes and cancer, which can be attributed to the presence of phytochemicals such as cinnamaldehyde, eugenol, trans-cinnamic acid, flavonoids and phenolic acids (3, 32). As cinnamaldehyde is the dominant bioactive compound accounting for >90% in cinnamon leaves, its protective role in the prevention of various chronic diseases has been extensively studied. For instance, for neuroprotection of Alzheimer’s disease, Peterson et al. reported that the aqueous extract of cinnamon from Ceylon was efficient in inhibiting tau protein aggregation, neurofibrillary tangles and amyloid beta plaques *in vitro* (5). In another study dealing with the neuroprotective effect of cinnamic aldehyde in a MPTP-induced mouse model of PD, Bae et al. (6) demonstrated that cinnamic aldehyde was protective against dopaminergic neuron loss and defective autophagy, which can lead to mitochondrial dysfunction and a substantial decrease in dopamine levels. Nevertheless, most published reports focused on the neuroprotective effects of cinnamaldehyde standard or solvent extracts from cinnamon tree bark, the possible treatment of PD by nanoemulsion prepared from cinnamon leaves remain less explored.

### 3.3. Characteristics of cinnamon leaf nanoemulsion

**Figure 2:**
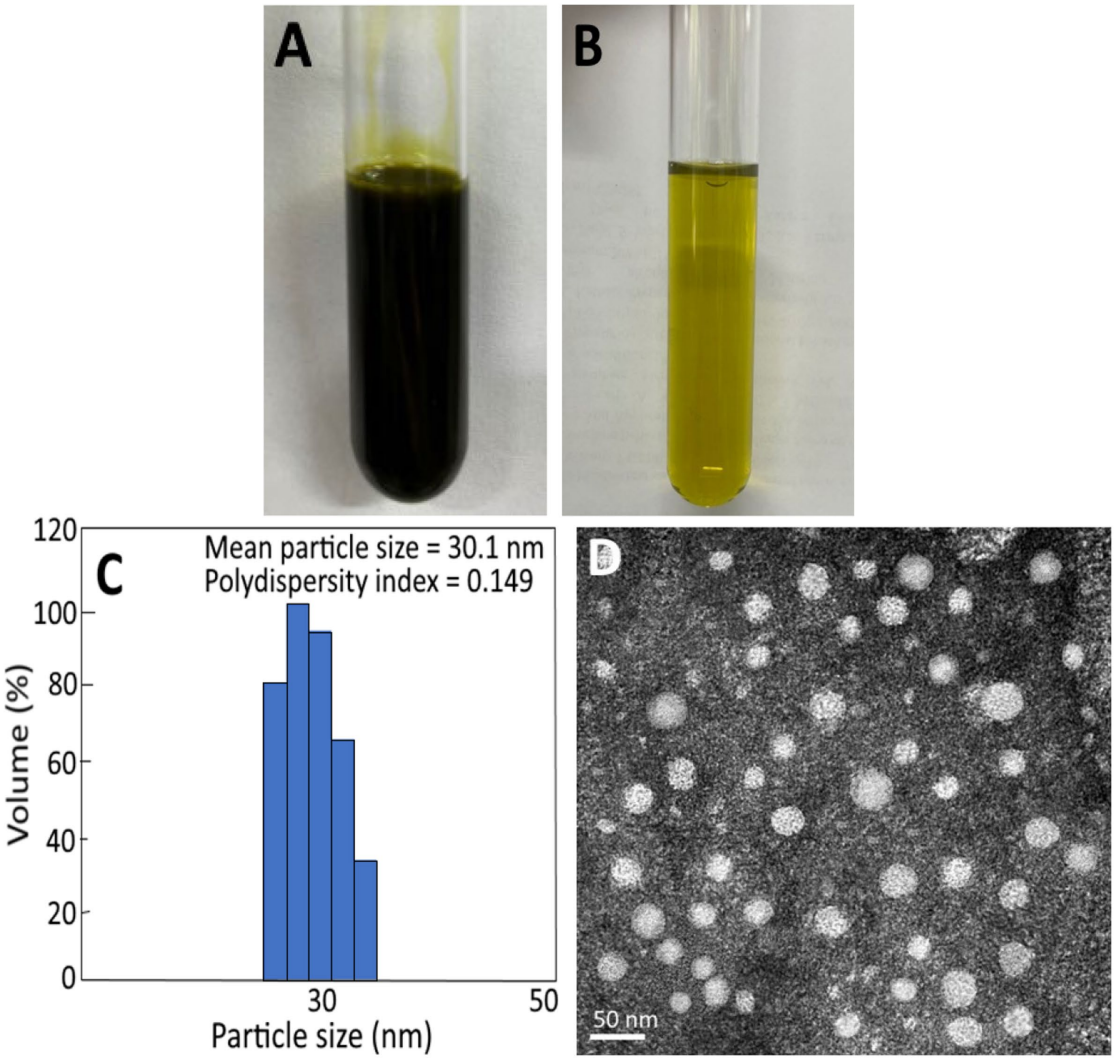
Appearance of cinnamon leaf nanoemulsion **(A)** and diluted cinnamon leaf nanoemulsion **(B)**, as well as, particle size distribution and polydispersity index as determined by dynamic light scattering (DLS) method **(C)** and transmission electron microscopic (TEM) image of cinnamon leaf nanoemulsion with mean particle size at 30 nm **(D)**.

As indicated above, cinnamaldehyde is the most abundant bioactive compound in cinnamon leaves. But due to poor aqueous solubility of cinnamaldehyde, its bioactivity *in vivo* may be affected. Thus, in this study we prepared cinnamon leaf nanoemulsion for encapsulation of cinnamaldehyde to enhance its stability *in vivo*. Figure 2A shows a deep green appearance of cinnamon leaf nanoemulsion, but following dilution, a transparent green color was shown in Figure 2B. The mean particle size of cinnamon leaf nanoemulsion was 30.1 nm and 30 nm as determined by DLS and TEM (Figures 2C,D), respectively. In addition, the PDI and zeta-potential was 0.149 and −43.1 mV, respectively, implying that a narrow and even distribution of nanoparticles in cinnamon leaf nanoemulsion, as well as, a high stability of this nanoemulsion was obtained. Based on a report by Lakshmi and Kumar (33), a PDI value between 0.1 and 0.25 indicated a narrow and even distribution of particles in solution and a PDI value of >0.5 indicated uneven and wide distribution. For zeta potential, a value of −43.1 mV implied a strong repulsive force between nanoparticles which is essential to impart high stability, as a zeta potential value ranging from >30 mV or <−30 mV represent a highly stable nanosystem (34). Moreover, this nanoemulsion can also exhibit high stability *in vivo*, as a high encapsulation efficiency (91.6%) was shown for cinnamaldehyde.

In several previous studies Mukerjee et al. (35) prepared a nanoemulsion by using cinnamon oil, usnic acid, Tween 80, ethanol and deionized water with a mean particle size of 96.39 nm and PDI of 0.25. Similarly, a mean particle size of 65 nm and PDI of 0.136 was found for the nanoemulsion prepared by using cinnamon oil, Tween 80 and deionized water (36). However, a much smaller particle size (8.69 nm) and PDI (0.22) was reported for nanoemulsion prepared with cinnamon oil, Tween 80 and clove extract (37), which can be attributed to addition of a high level of Tween 80. Nevertheless, the incorporation of high-level Tween 80 into a nanoemulsion may elevate health risk. In our study we also used lecithin as a surfactant to enhance the stability of the o/w nanoemulsion and thus the amount of Tween 80 addition was reduced. In another study Dávila-Rodríguez et al. (38) prepared 3 nanoemulsions from cinnamon, rosemary and oregano essential oils and the mean particle size was shown to be, respectively, 176, 267, and 414 nm, and the encapsulation efficiency 98.9, 97.0, and 82.8%, while the mean particle size and encapsulation efficiency of a similar cinnamon nanoemulsion was reported to be 100 nm and 81%, respectively (39). Apparently the difference in particle size, PDI, zeta potential and encapsulation efficiency of cinnamon nanoemulsions can be driven by variety and amount of ingredients used for nanoemulsion preparation.

Tables 3, 4 show the storage and heat stability data of cinnamon leaf nanoemulsion respectively, revealing a high stability of this nanoemulsion prepared in our study as only a minor change in mean particle size, PDI and zeta potential was observed over a 90 days storage period at 4°C and heating for 2 h. However, we have to point out that after prolonged heating at high temperature, the turbid appearance of this nanoemulsion can occur and then revert back to original state following cooling and stirring. This phenomenon may be caused by flocculation due to interaction between particles for formation of irregular aggregates. Nevertheless, a high level of cinnamaldehyde in the nanoemulsion may also cause this phenomenon as evident by formation of a transparent green appearance after dilution (Figure 2B). Moreover, it is possible that the surfactant solubility in nanoemulsion follows a temperature-dependent rise during heating, however, when the temperature exceeds cloud point, the solubility declines to lower the stability of nanoemulsion and results in a turbid appearance, which can revert back to the original transparent appearance after the temperature decreases to be below cloud point (40). As no irreversible change occurs for this nanoemulsion during 90 days storage at 4°C, this outcome demonstrates a high storage stability of cinnamon leaf nanoemulsion prepared in our study.

**Table 3:** Particle size and zeta-potential changes of cinnamon leaf nanoemulsion during storage for 90 days at 4°C.

Day	Particle size (nm)^a^	Polydispersity index^b^^,^^c^	Zeta-potential (mV)^b^^,^^c^
0	30.1	0.149 ± 0.02^a^	−43.1 ± 0.77^d^
7	32.3	0.175 ± 0.03^a^	−35.6 ± 0.70^a^
14	32.1	0.176 ± 0.01^a^	−35.1 ± 1.50^a^
21	30.7	0.168 ± 0.06^a^	−37.6 ± 0.71^b^
30	28.9	0.156 ± 0.02^a^	−35.3 ± 0.06^a^
60	27.9	0.148 ± 0.04^a^	−38.3 ± 0.68^b^
90	30.5	0.186 ± 0.01^a^	−41.3 ± 1.05^c^

a Data shown are mean of triplicate analyses.

b Data shown are mean ± standard deviation of triplicate analyses.

c Data with different small letters (a)–(d) in the same column are significantly different at *p* < 0.05.

**Table 4:** Particle size and zeta-potential changes of cinnamon leaf nanoemulsion during heating at 40°C, 70°C, and 100°C for varied time length.

Temperature (°C)	Particle size (nm)					Polydispersity index					Zeta-potential (mV)				
Time length^a^	0 h	0.5 h	1 h	1.5 h	2 h	0 h	0.5 h	1 h	1.5 h	2 h	0 h	0.5 h	1 h	1.5 h	2 h
Control	30.1	–	–	–	–	0.149	–	–	–	–	−43.1	–	–	–	–
40	–	28.9	26.6	26.9	25.3	–	0.159	0.179	0.155	0.152	–	−41.3	−40.7	−40.6	−40.3
70	–	25.6	26.1	26.4	24.8	–	0.175	0.166	0.185	0.173	–	−39.9	−39.3	−39.2	−38.2
100	–	25.2	24.6	23.7	24.4	–	0.187	0.165	0.176	0.153	–	−38.1	−37.5	−36.1	−35.0

a Data shown are mean of triplicate analyses.

### 3.4. Animal study

**Figure 3:**
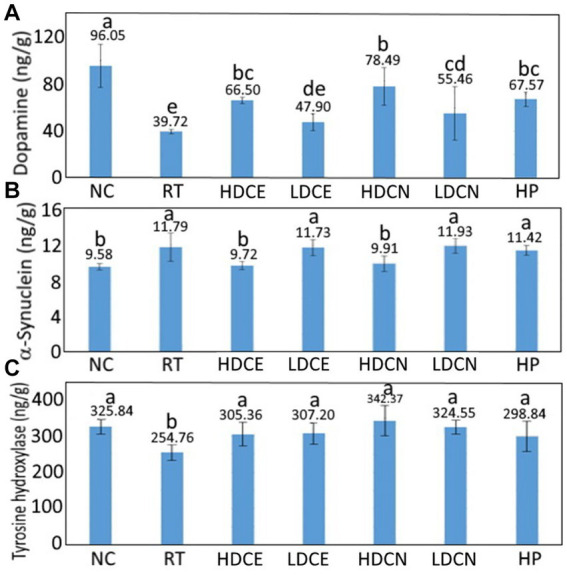
Effects of cinnamon leaf extract, nanoemulsion and powder hydrosol on dopamine **(A)**, α-synuclein **(B)**, and tyrosine hydroxylase **(C)** contents in striatum of rotenone-induced Parkinson’s disease (PD) rats. Data are presented as mean ± standard deviation (*n* = 6). NC, normal control group; RT, rotenone (2 mg/kg bw) dissolved in sunflower oil and administered daily for a period of 5 weeks (35 days); High-dose cinnamon extract (HDCE), administration of cinnamon leaf extract at a cinnamaldehyde dose of 60 mg/kg bw and rotenone (2 mg/kg bw) for 35 days; Low-dose cinnamon extract (LDCE), administration of cinnamon leaf extract at a cinnamaldehyde dose of 20 mg/kg bw and rotenone (2 mg/kg bw) for 35 days; High-dose cinnamon nanoemulsion (HDCN), administration of cinnamon leaf nanoemulsion at a cinnamaldehyde dose of 60 mg/kg bw and rotenone (2 mg/kg bw) for 35 days; Low-dose cinnamon nanoemulsion (LDCN), administration of cinnamon leaf nanoemulsion at a cinnamaldehyde dose of 20 mg/kg bw and rotenone (2 mg/kg bw) for 35 days; Powder in hydrosol (HP), administration of leaf powder dissolved in hydrosol (0.5 g/10 mL) at a dose of 10 mL/kg bw and rotenone (2 mg/kg bw) for 35 days; bw, body weight. Data are presented as mean ± standard deviation (*n* = 8) and data with different small letters **(a)–(e)** are significantly different at *p* < 0.05.

Figure 3A shows the effect of various treatments on dopamine contents in the striatum of the rotenone-induced PD rats. Rotenone, a hydrophorbic neurotoxin, can cross BBB readily through subcutaneous injection without brain opening and transportation through a dopamine transporter. Furthermore, rotenone was capable of inhibiting activity of mitochondria complex I and leading to production of free radicals and dysfunction of mitochondria for subsequent loss of dopaminergic neuron (41). Compared to the rotenone-induced (RT) group, the dopamine content for the control (NC) group was significantly higher (*p* < 0.05) by 56.33 ng/g (58.64%), while for the HDCE, LDCE, HDCN, LDCN, and HP groups, the dopamine contents were raised by 26.78 ng/g (40.27%), 8.18 ng/g (17.08%), 38.77 ng/g (49.39%), 15.74 ng/g (28.38%), and 27.85 ng/g (41.22%). Thus, the HDCN was the most effective in raising dopamine contents in the striatum in the rotenone-induced PD rats.

Dopamine was analyzed as PD is a movement disorder disease caused by degeneration of brain cells of the basal ganglia and substantia nigra for inadequate production of dopamine as mentioned above. This symptom occurs when the dopamine level secreted is lower than 80% of normal. Additionally, the striatum in the human midbrain possesses the function of regulating muscle tension and coordinating complex movements, both of which need to rely on dopamine to work normally (17). Therefore, the presence of dopamine level in the striatum is a vital index of PD.

In several similar studies the administration of PD rats with cinnamon powder for 7 days was shown to raise the dopamine content in the striatum by 35% (42), which can be ascribed to the presence of high amount of cinnamaldehyde in cinnamon powder. In another study investigating the effect of eugenol on dopamine contents in the striatum in 6-OHDA induced PD rats, a significant rise (*p* < 0.05) of 56.5, 54.5, and 44.4% was observed for the treatments of two doses (1.0 and 0.1 mmol/kg) before induction and one dose (1.0 mmol/kg) after induction, respectively. This outcome also implied that in addition to cinnamaldehyde, eugenol also plays a critical role in improving PD in rats. In addition to cinnamaldehyde and eugenol, the effect of flavonoids such as quercetin and phenolic acids such as caffeic acid on improving PD syndrome in rats cannot be ignored. For instance, compared to the 6-OHDA induced group, the dopamine content in the striatum of PD rats was raised by 34.56% following intake of quercetin at 30 mg/kg for 14 days (43). Similarly, after intraperitoneal injection of PD rats with quercetin at 25, 50, and 75 mg/kg for 4 days, the dopamine contents in the striatum rose by 9, 25, and 39%, respectively, when compared to the induction group (44). In a later study Ablat et al. (45) further demonstrated that kaempferol-3-O-rutiniside, the major flavonoid in safflower, was effective in increasing the dopamine contents in the striatum of PD rats by 27 and 25%, respectively, after tube feeding with the safflower extract at 70 and 35 mg/kg for 24 days. More recently, Kumar et al. (46) prepared a fisetin nanoemulsion with the mean particle size being 154 nm for subsequent tube feeding at 20 mg/kg (high-dose fisetin), 10 mg/kg (low-dose nanoemulsion), and 20 mg/kg (high-dose nanoemulsion) into PD rats for 35 days, the dopamine contents in the striatum rose by 35, 42, and 50%, respectively, when compared to the induction group. Compared to most published reports, the dopamine contents in PD rats were higher for the high-dose cinnamon leaf nanoemulsion (raised by 49.39%) in our study, probably due to the presence of much smaller particle size (30.1 nm) in this nanoemulsion, making it more susceptible to cross BBB. The cross efficiency of nanoparticles into BBB has been controversial. In a previous International Nanomedicine Workshop held at London, United Kingdom, Dawson (47) reported that nanoparticles with size <100 nm can enter into cells, while the size <40 nm can enter into nucleus and that <35 nm can cross BBB. Tang et al. (48) studied the effect of silver nanoparticle on BBB and reported that the smaller the particle size, the higher the content of silver nanoparticles in brain. In another study Betzer et al. (49) compared the effect of nanoparticle size (nanogold conjugated with insulin) on the ability to cross BBB in mice, a size of 20 nm nanogold was the most widely distributed in brain compared to the nanogold with a size of 50 or 70 nm, probably caused by conjugation of nanogold insulin with insulin receptor in brain cells. Interestingly, in a report investigating the effect of nanoparticle size (nanogold) on the delivery into brain assisted by focused ultrasound-induced BBB opening, a size of 15 nm was the most effective in crossing BBB, followed by 3 nm and 120 nm (50). It may be inferred that the smallest nanogold size (3 nm) should be the most efficient in crossing BBB, however, it may also undergo a fast kidney excretion. Thus, the cross efficiency into BBB as affected by nanoparticle size needs to be further explored. Nevertheless, the nanoemulsion size prepared in our study is small (30 nm), which should be able to cross BBB to activate brain cells for secretion of more dopamine in improving PD syndrome in rats.

Furthermore, it has been well established that BBB can inhibit entrance of about 98% of small-molecule drugs and 100% of large-molecule drugs into brain, but with the small size nature and capability of encapsulating drugs of nanoparticles, it is possible that nanoparticles can cross BBB in a more efficient way (51, 52). Of the various nanosystems, the nanoemulsion (o/w) has been shown to be able to enhance aqueous solubility and encapsulation of lipophilic drugs for prevention of enzyme degradation *in vivo* (53). In addition, it was reported that the surfactant Tween 80 used for preparing nanoemulsion in our study could facilitate ApoE adsorption from circulation for conjugation with the LDL receptor of brain cell membrane for easier penetration into BBB, and for subsequent uptake of nanoparticles by brain endothelial cells (54–56).

The α-synuclein levels in the striatum of rats as affected by various treatments are also shown in Figure 3B. Compared to the RT group, the α-synuclein level was lowered by 23.06% for the NC group, but for the HDCE, LDCE, HDCN and HP groups, the α-synuclein levels were reduced by 17.56, 0.50, 15.95, and 3.10%, respectively. This outcome indicated that both high-dose cinnamon leaf extract and nanoemulsion were the most effective in decreasing α-synuclein levels in the striatum of rats.

Synuclein, including α-, β-, and γ-synuclein, is a small soluble protein family, in which α-synuclein is a relatively small and soluble protein which can be expressed presynaptically and perinuclearly in the central nervous system for regulation of neuronal stability, presynaptic signaling and membrane trafficking of vesicles (17). However, α-synuclein can undergo aggregation to form Lewy bodies, which in turn cause PD, Lewy’s dementia and multiple system atrophy. In addition, various cellular diseases such as oxidative stress, synaptic and mitochondrial dysfunction, dysregulation of calcium signaling and microvascular damage can occur (17). Thus, the determination of α-synuclein level in the striatum is imperative.

In a similar study Raha et al. (57) studied the effect of cinnamon on α-synuclein aggregation in transgenic mice (A53T) and reported that the α-synuclein level was reduced by 66% after tube feeding of cinnamon powder at 100 mg/kg for 60 days. In addition to cinnamon leaf, the effect of some other plants rich in phenolic acids and flavonoids on α-synuclein levels in mice/rats has been reported. For example, the *Cyanara scoluymus* extract was effective in diminishing α-synuclein levels in PD rats by 73.90% after tube feeding at 200 mg/kg for 20 days. Likewise, after tube feeding of PD mice with *Tribulus terrestris* extract at 100, 300, and 1,000 mg/kg for 21 days, the α-synuclein levels were decreased by 27.8, 44.0, and 52.0%, respectively (58). A similar outcome was reported by Tikhonova et al. (59) and Guo et al. (60), with the former showing a reduction of α-synuclein level in transgenic mice A53T by 50% after tube feeding with grape polyphenol at 1.5 mL/kg for 4 months, and the latter showing a decline of 78.5% in PD mice after tube feeding with resveratrol at 100 mg/kg/d, when compared to the induction group. Collectively, the presence of bioactive compounds such as cinnamaldehyde, phenolic acids and flavonoids were effective in diminishing α-synuclein levels in the striatum of PD mice/rats. However, compared to most published reports, a less reduction of α-synuclein levels in PD rats was observed in our study, probably due to the difference in animal strain, variety and amount of bioactive compounds, feeding dose and period, and induction mode.

The effect of various treatments on tyrosine hydroxylase contents in the striatum in PD rats is shown in Figure 3C. Compared to the RT group, the tyrosine hydroxylase content was higher by 21.81% for the NC group, as well as, higher by 16.57, 17.07, 25.59, 21.50, and 14.75% for the HDCE, LDCE, HDCN, LDCN, and HP groups, respectively. This result indicated that the high-dose nanoemulsion was the most effective in elevating tyrosine hydroxylase content. Tyrosine hydroxylase in the striatum needs also to be analyzed as it can convert tyrosine into L-dopamine in human body. Specifically, the lack of tyrosine hydroxylase can cause inadequate production of dopamine due to neurotransmitter blockage in the central and peripheral nervous system.

In several similar studies, after intraperitoneal injection of cinnamaldehyde at 10 mg/kg into mice for 1 week, the tyrosine hydroxylase level was raised by 50% (6), while a rise by 38.11% was also shown in PD mice following intraperitoneal injection of cinnamaldehyde at 30 mg/kg for 48 h (61), when compared to the induction group. In addition to cinnamaldehyde, the tyrosine hydroxylase level was raised by 68.7% after tube feeding of PD rats with safflower extract at 70 mg/kg for 24 days (45), while a mounting amount by 42.8% was found in PD mice after tube feeding with resveratrol at 100 mg/kg/d for 33 days (60), when compared to the induction group. Apparently, the presence of cinnamaldehyde and the other bioactive compounds such as phenolic acids and flavonoids may play a vital role in elevating tyrosine hydroxylase contents in PD rats/mice. However, in our study the tyrosine hydroxylase level was less reduced compared to published reports, which may be due to the difference in the variety and amount of sample, feeding period, animal strain and feeding dose.

**Figure 4:**
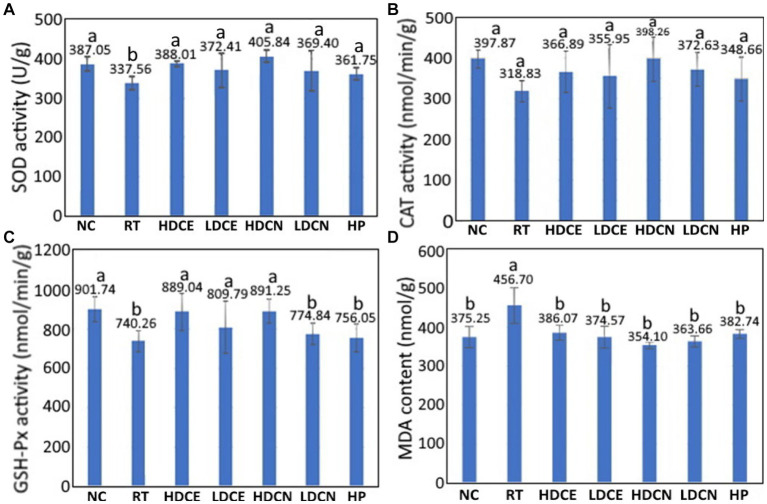
Effects of cinnamon extract, nanoemulsion and powder hydrosol on superoxide dismutase (SOD) **(A)**, catalase (CAT) **(B)**, glutathione peroxidase (GSH-Px) **(C)**, and malondialdehyde (MDA) **(D)** contents in midbrain of rotenone-induced PD rats. NC, normal control group; RT, rotenone (2 mg/kg bw) dissolved in sunflower oil and administered daily for a period of 5 weeks (35 days); High-dose cinnamon extract (HDCE), administration of cinnamon leaf extract at a cinnamaldehyde dose of 60 mg/kg bw and rotenone (2 mg/kg bw) for 35 days; Low-dose cinnamon extract (LDCE), administration of cinnamon leaf extract at a cinnamaldehyde dose of 20 mg/kg bw and rotenone (2 mg/kg bw) for 35 days; High-dose cinnamon nanoemulsion (HDCN), administration of cinnamon leaf nanoemulsion at a cinnamaldehyde dose of 60 mg/kg bw and rotenone (2 mg/kg bw) for 35 days; Low-dose cinnamon nanoemulsion (LDCN), administration of cinnamon leaf nanoemulsion at a cinnamaldehyde dose of 20 mg/kg bw and rotenone (2 mg/kg bw) for 35 days; Powder in hydrosol (HP), administration of leaf powder dissolved in hydrosol (0.5 g/10 mL) at a dose of 10 mL/kg bw and rotenone (2 mg/kg bw) for 35 days; bw, body weight. Data are presented as mean ± standard deviation (*n* = 8) and data with different small letters **(a)** and **(b)** are significantly different at *p* < 0.05.

As reactive oxygen species (ROS) have been demonstrated to be a vital factor in causing neurodegenerative disease such as PD, the effect of various treatments on activities of antioxidant enzyme in the midbrain of PD rats needs to be explored. The SOD activity in the midbrain of PD rats as affected by various treatments is shown in Figure 4A. Compared to the RT group, the SOD activity for the NC group was significantly higher (*p* < 0.05) by 12.79%. Similarly, for the HDCE, LDCE, HDCN, LDCN and HP groups, the SOD activities rose by 13.00, 9.36, 16.82, 8.62, and 6.69%, respectively, implying that the high-dose nanoemulsion showed the most prominent effect in enhancing SOD activity.

An analogues trend was found for the CAT activity as illustrated in Figure 4B. Compared to the RT group, the CAT activities for the NC, HDCE, LDCE, HDCN, LDCN, and HP groups were raised by 19.87, 9.16, 10.43, 19.94, 14.43, and 8.56%, respectively, revealing that the high-dose nanoemulsion was the most efficient in increasing CAT activity. Likewise, for the GSH-Px activity shown in Figure 4C, it was increased by 17.91, 16.73, 8.59, 16.94, 4.46, and 2.09% for the NC, HDCE, LDCE, HDCN, LDCN and HP groups, respectively. By comparison, both high-dose extract and nanoemulsion showed the most pronounced effect in elevating the GSH-Px activity in PD rats. On the contrary, a different result was observed for the MDA content, a degradation product formed from linolenate hydroperoxide during thermal oxidation of oil, which can be used to assess the degree of lipid oxidation. The MDA content changes as affected by various treatments are also shown in Figure 4D. Compared to the RT group, the MDA contents in rat midbrain were reduced by 17.83, 15.47, 17.98, 22.47, 20.37, and 16.19% for the groups of NC, HDCE, LDCE, HDCN, LDCN, and HP, respectively, with the high-dose nanoemulsion being the most effective in lowering MDA contents in PD rats. Obviously, the increase of antioxidant enzyme activity and decrease of MDA contents in midbrain of PD rats can be due to the presence of bioactive compounds such as cinnamaldehyde, phenolic acids and flavonoids in cinnamon leaves. In several previous studies Mehraein et al. (62) reported that compared to the induction group, the CAT activity rose by 42% while the MDA level reduced by 32% in the midbrain of PD rats following intraperitoneal injection of cinnamaldehyde oil at 30 mg/kg into MPTP-induced rats for 2 weeks. In a study reporting the effect of gold-cinnamon leaf nanoemulsion (30–50 nm) on neuroprotection of PD rats, Ling et al. (63) demonstrated that compared to the induction group, the SOD activity increased by 25.9 and 37.5%, respectively, after administration of this nanoemulsion at 5 and 10 mg/kg for 15 days. In addition to cinnamaldehyde, eugenol, one of the main bioactive compounds in cinnamon leaves, was also shown effective in reducing the MDA content by 34.4% in PD rats after intraperitoneal injection at 1.0 or 0.1 mmol/kg for 7 days (64). Also, the effects of plant extract nanoemulsion rich in phenolic acids and flavonoids on improving antioxidant enzyme activities in PD rats were investigated by several authors. For instance, compared to the induction group, the SOD, CAT, and GSH-Px activities rose by 68.1, 15.0, and 25.5%, respectively, while the MDA content decreased by 38.9%, after administration of PD rats with *Tribulus terrestris* extract at 1000 mg/kg for 21 days (58), which may be due to kaempferol being present in high amount.

In a comparative study on neuroprotection of nerve-damaged and movement disordered rats, the SOD activities, respectively, rose by 26.2 and 33.6% and the MDA contents declined by 20.1 and 25.6% after administration with rutin extract and nanoemulsion (65) Likewise, Kumar et al. (66). prepared fisetin nanoemulsion and reported that the high-dose nanoemulsion was the most effective in diminishing MDA contents in PD rats by 72.3%, followed by high-dose extract (65.2%), low-dose nanoemulsion (55.8%) and low-dose extract (43.3%). More recently, the high-dose curcumin nanoemulsion was shown to be the most efficient in lowering the MDA content in PD rats by 75.4%, followed by low-dose nanoemulsion (73.1%), high-dose curcumin extract (62.6%) and low-dose curcumin extract (52.7%) (67). All the results shown above are in agreement with our finding that the nanoemulsion is superior to the extract in improving PD in rats through elevation of the antioxidant enzyme activity and decline in MDA content.

### 3.5. Catalepsy test

**Figure 5:**
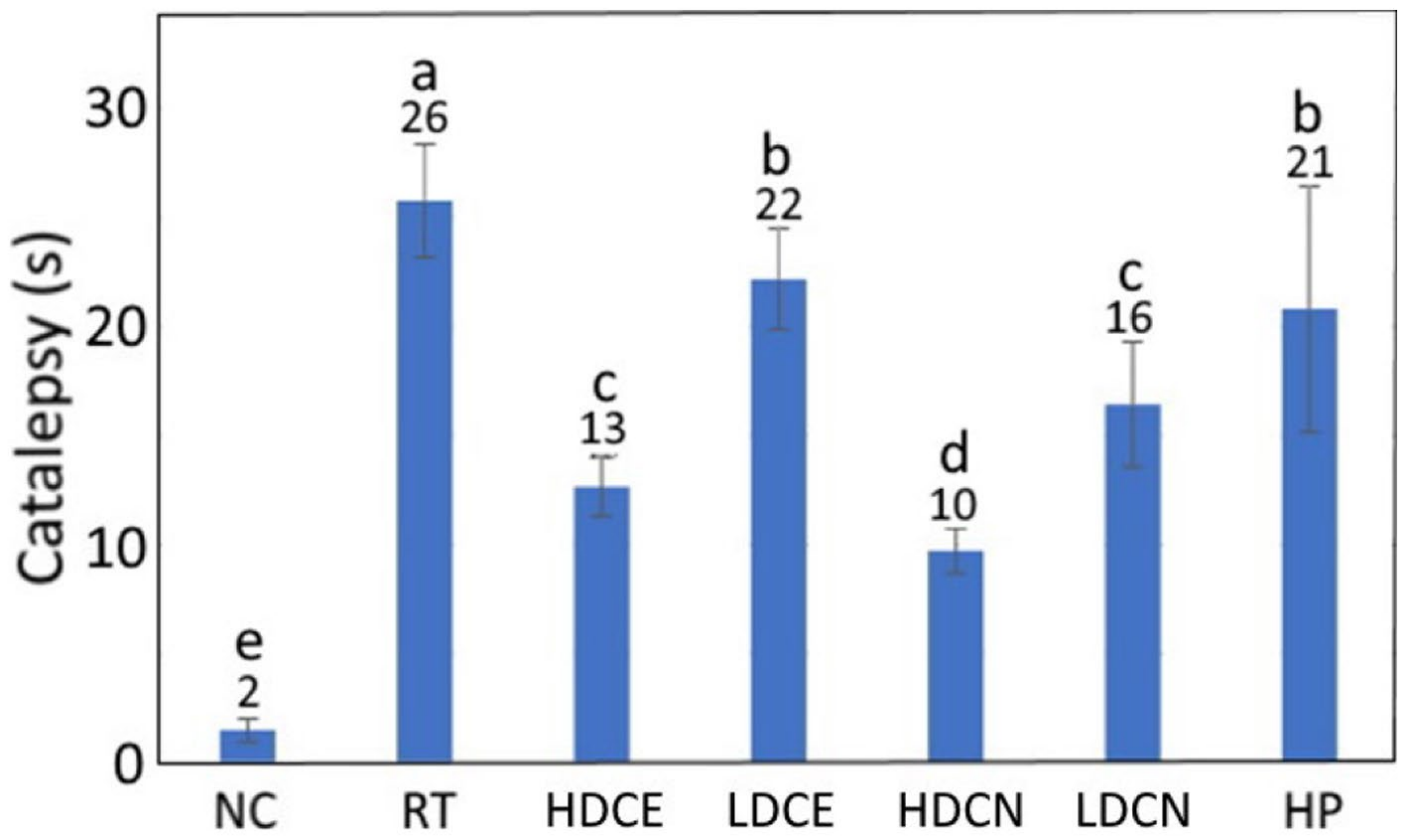
Effects of cinnamon leaf extract, nanoemulsion, and powder hydrosol on catalepsy of rotenone-induced PD rats. NC, normal control group; RT, rotenone (2 mg/kg bw) dissolved in sunflower oil and administered daily for a period of 5 weeks (35 days); High-dose cinnamon extract (HDCE), administration of cinnamon leaf extract at a cinnamaldehyde dose of 60 mg/kg bw and rotenone (2 mg/kg bw) for 35 days; Low-dose cinnamon extract (LDCE), administration of cinnamon leaf extract at a cinnamaldehyde dose of 20 mg/kg bw and rotenone (2 mg/kg bw) for 35 days; High-dose cinnamon nanoemulsion (HDCN), administration of cinnamon leaf nanoemulsion at a cinnamaldehyde dose of 60 mg/kg bw and rotenone (2 mg/kg bw) for 35 days; Low-dose cinnamon nanoemulsion (LDCN), administration of cinnamon leaf nanoemulsion at a cinnamaldehyde dose of 20 mg/kg bw and rotenone (2 mg/kg bw) for 35 days; Powder in hydrosol (HP), administration of leaf powder dissolved in hydrosol (0.5 g/10 mL) at a dose of 10 mL/kg bw and rotenone (2 mg/kg bw) for 35 days; bw, body weight. Data are presented as mean ± standard deviation (*n* = 8) and data with different small letters **(a)**–**(e)** are significantly different at *p* < 0.05.

The catalepsy test data as affected by various treatments is depicted in Figure 5. Compared to the RT group, the catalepsy time was reduced by 92.31, 42.31, 15.38, 61.54, 38.46, and 19.23% for the CD, HDCE, LDCE, HDCN, LDCN, and HP groups, respectively. Comparatively, with the exception of CD, the high-dose nanoemulsion was the most effective in decreasing catalepsy time of PD rats. Nevertheless, a paucity of data does exist to demonstrate the effect of cinnamon extract and nanoemulsion on reduction of catalepsy time in PD rats. In a recent study Churihar et al. (68) evaluated the effect of cinnamaldehyde on haloperidol induced catalepsy in albino mice and reported that following tube feeding of cinnamaldehyde at two doses (100 and 200 mg/kg), the catalepsy time remained unaffected, probably caused by the cinnamaldehyde instability *in vivo* and lack of some other bioactive compounds such as phenolic acids and flavonoids. Several other studies dealing with the effect of plant extracts rich in phenolic acids and flavonoids on improving catalepsy time in PD rats have been reported. For example, compared to the induction group, the catalepsy time declined by 88.6% after tube feeding of PD rats with *Cynara scolymus* (artichoke) at 200 mg/kg for 20 days (69). Similarly, following administration of PD mice with *Tribulus terrestris* (caltrop) extract at 100, 300, and 1,000 mg/kg for 21 days, the catalepsy time was lowered by 54.6, 54.6, and 81.9%, respectively (58). In a study dealing with the effect of fisetin extract and nanoemulsion on improving catalepsy time in PD rats after tube feeding of two doses (10 and 20 mg/kg) for 35 days, the high-dose nanoemulsion was the most efficient in reducing catalepsy time by 32.9%, followed by low-dose nanoemulsion (29.9%), high-dose extract (20.4%) and low-dose extract (11.45%) (66). A similar outcome was reported for rutin extract and nanoemulsion as evidenced by a reduction of catalepsy time by 38.8 and 50.6%, respectively, in nerve-damaged rats when compared to the induction group (65). These results are in accordance with our finding, demonstrating that the nanoemulsion was superior to the extract in improving catalepsy performance, probably due to enhanced protection of bioactive compounds such as cinnamaldehyde, phenolic acids and flavonoids in the nanoemulsion through encapsulation.

The foregoing discussion demonstrated the efficiency of high-dose cinnamon leaf nanoemulsion in improving PD in rats and thus can be further evaluated through clinical trials for patients with PD. However, prior to *in vivo*/human trials, verifying the efficiency of cinnamon leaf nanoemulsion by a preclinical model is pivotal, as the new law enforced in December 2022 states that FDA no longer requires animal testing for new drugs and can rely on *in vitro* experiment before conducting clinical trial studies (70). From the brain structure point of view, this is particularly important as the BBB severely hinders drug access to CNS and the global burden of neurological disorders has increased substantially over the past two decades (71, 72). Consequently, researchers have attempted to develop reliable *in vitro* models to screen the ability of novel drug delivery systems to cross the BBB, thereby preventing the therapeutic inefficiency of drugs during *in vivo* and clinical trial studies. Many cell-based *in vitro* models have been developed to determine the permeability of drugs into the BBB, with the Transwell models being the most widely applied cell-based *in vitro* BBB models (73). But these 2D *in vitro* models fail to fully reproduce the complexity of human BBB, resulting in the inability to accurately predict the *in vivo*/clinical permeation. Therefore, for effective clinical translation from bench to bedside, a therapeutic drug requires to be screened by robust and physiologically relevant microvascular 3D *in vitro* models and some recent reviews have comprehensively elucidated the emerging 3D vitro models including hydrogel-, spheroid- and organoid-based static BBB models as well as microfluidic-based BBB-on-a-chip models highlighting their application, advantages and disadvantages in screening drugs (73, 74). Thus, a future study is warranted to evaluate the therapeutic efficiency of cinnamon leaf nanoemulsion by a 3D *in vitro* BBB model prior to clinical trial experiments.

## 4. Conclusion

In conclusion, an UPLC-MS/MS method with ESI mode was employed to separate, identify and quantify 15 bioactive compounds in cinnamon leaves, with cinnamaldehyde being the dominant compound (17985.2 μg/g). The nanoemulsion composed of soybean oil, lecithin, Tween 80 and deionized water was successfully prepared with a mean particle size of 30.1 nm, PDI of 0.149, zeta potential of −43.1 mV and encapsulation efficiency of 91.6%. Additionally, a high storage ability (4°C for 3 mo) and heating stability (100°C for 2 h) was observed. Of the various treatments, the high-dose nanoemulsion showed the most pronounced effect in elevating both dopamine and tyrosine hydroxylase contents, as well as, the antioxidant enzyme activities including SOD, CAT and GSH-Px, while decreasing 𝛼-synuclein aggregation and MDA contents. Taken together, the high-dose nanoemulsion showed the most pronounced effect in improving Parkinson’s disease in rats and should possess a great potential to be developed into a health food or botanic drug. Nevertheless, a future study is warranted to evaluate the cinnamon leaf nanoemulsion by a 3D *in vitro* BBB model prior to testing their therapeutic potential through clinical trials for patients with Parkinson’s disease.

## Data availability statement

The original contributions presented in the study are included in the article/Supplementary material, further inquiries can be directed to the corresponding author.

## Ethics statement

The animal study was reviewed and approved by Fu Jen University Animal Care and Use Committee.

## Author contributions

YW: methodology, investigation, formal analysis, software, data curation, validation, and writing – original draft. VW: investigation, formal analysis, data curation, validation, visualization, and resources. BC: conceptualization, methodology, resources, validation, visualization, writing – original draft, writing – review and editing, project administration, supervision, and funding acquisition. All authors contributed to the article and approved the submitted version.

## Funding

The authors declare that this study received funding from Tou-Fu Investment Co, Taipei, Taiwan (Grant no. 7100339). The funder was not involved in the study design, collection, analysis, interpretation of data, the writing of this article, or the decision to submit it for publication.

## Conflict of interest

The authors declare that the research was conducted in the absence of any commercial or financial relationships that could be construed as a potential conflict of interest.

## Publisher’s note

All claims expressed in this article are solely those of the authors and do not necessarily represent those of their affiliated organizations, or those of the publisher, the editors and the reviewers. Any product that may be evaluated in this article, or claim that may be made by its manufacturer, is not guaranteed or endorsed by the publisher.

## Supplementary material

The Supplementary material for this article can be found online at: https://www.frontiersin.org/articles/10.3389/fnut.2023.1229192/full#supplementary-material

## References

[1] Chen C Chen FA. Research status of indigenous cinnamon (Cinnamomum osmophloeum Kanehira). Tajen J. (2018) 52:1–27.

[2] Lee SC Xu W Lin L Yang JJ Liu CT. Chemical composition and hypoglycemic and pancreas-protective effect of leaf essential oil from indigenous cinnamon (Cinnamomum osmophloeum kanehira). J Agric Food Chem. (2013) 61:4905–13. doi: 10.1021/jf401039z, PMID: 23627599

[3] Błaszczyk N Rosiak A Kałużna-Czaplińska J. The potential role of cinnamon in human health. Forests. (2021) 12:648. doi: 10.3390/f12050648, PMID: 35792680

[4] Huang YC Chen BH. A comparative study on improving streptozotocin-induced type 2 diabetes in rats by hydrosol, extract and nanoemulsion prepared from cinnamon leaves. Antioxidants. (2023) 12:29. doi: 10.3390/antiox12010029 PMC9855112 36670891

[5] Peterson DW George RC Scaromozzino F LaPointe NE Anderson RA Graves DJ. Cinnamon extract inhibits tau aggregation associated with alzheimer’s disease in vitro. J Alzheimers Dis. (2009) 17:585–97. doi: 10.3233/JAD-2009-1083, PMID: 19433898

[6] Bae WY Choi JS Jeong JW. The neuroprotective effects of cinnamic aldehyde in an MPTP mouse model of Parkinson’s disease. Int J Mol Sci. (2018) 19:551. doi: 10.3390/ijms19020551, PMID: 29439518 PMC5855773

[7] Ye AY. Nanotechnology and food. Sci Dev. (2007) 418:42–7.

[8] Yu JX Li TH. Distinct biological effects of different nanoparticles commonly used in cosmetics and medicine coatings. Cell Biosci. (2011) 1:9. doi: 10.1186/2045-3701-1-19 21711940 PMC3125209

[9] Gupta PK Bhandari N Shah H Khanchandani V Keerthana R Nagarajan V. Nanoemulsions: formation, properties and applications. Soft Matter. (2019) 12:2826–41. doi: 10.1039/C5SM02958A 26924445

[10] Alexander A Agrawal M Uddin A Siddique S Shehata AM Shaker MA. CCCC recent expansions of novel strategies towards the drug targeting into the brain. Int J Nanomedicine. (2019) 14:5895–909. doi: 10.2147/IJN.S210876 31440051 PMC6679699

[11] Li D Wei Z Xue C. Alginate-based delivery systems for food bioactive ingredients: an overview of recent advances and future trends. Compr Rev Food Sci Food Saf. (2021) 20:5345–69. doi: 10.1111/1541-4337.12840, PMID: 34596328

[12] Mahfoudhi N Ksouri R Hamdi S. Nanoemulsions as potential delivery systems for bioactive compounds in food systems: preparation, characterization, and applications in food industry. Emulsions-nanotechnology in the Agri-food industry, 3, Elsevier, New York, USA, (2016), 365–403

[13] McClements DJ Rao J. Food-grade nanoemulsions: formulation, fabrication, properties, performance, biological fate, and potential toxicity. Crit Rev Food Sci Nutr. (2011) 51:285–330. doi: 10.1080/10408398.2011.559558, PMID: 21432697

[14] WHO. Neurological disorders: Public health challenges, (2020). Available at: https://www.who.int/mental_health/neurology/neurodiso/en.

[15] TFDA. Statistical report of Parkinson’s disease patients in Taiwan, Taipei, Taiwan: Taiwan Food and Drug Analysis Publishers. (2021).

[16] Liu CC Li CY Lee PC Sun Y. Variations in incidence and prevalence of PD's disease in Taiwan: a population-based nationwide study. Parkinsons Dis. (2016) 2016:8756359. doi: 10.1155/2016/8756359, PMID: 26904358 PMC4745820

[17] Wakhloo D Overhauser J Madira A Mahajani S. From cradle to grave: neurogenesis, neuroregeneration and neurodegeneration in Alzheimer’s and Parkinson’s diseases. Neural Regen Res. (2022) 17:2606–14. doi: 10.4103/1673-5374.336138, PMID: 35662189 PMC9165389

[18] Mani M Balasubramanian S Manikandan KR Kulandaivel B. Neuroprotective potential of naringenin-loaded solid-lipid nanoparticles against rotenone-induced Parkinson’s disease model. J Appl Pharm Sci. (2021) 11:19–28. doi: 10.7324/JAPS.2021.110203

[19] Sita G Hrelia P Tarozzi A Morroni F. Isothiocyanates are promising compounds against oxidative stress, neuroinflammation and cell death that may benefit neurodegeneration in PD's disease. Int J Mol Sci. (2016) 17:1454. doi: 10.3390/ijms17091454, PMID: 27598127 PMC5037733

[20] Kumar GP Khanum F. Neuroprotective potential of phytochemicals. Pharmacogn Rev. (2012) 6:81. doi: 10.4103/0973-7847.99898, PMID: 23055633 PMC3459459

[21] Mateen S Rehman M Shahzad S Naeem SS Faizy AF Khan AQ. Anti-oxidant and anti-inflammatory effects of cinnamaldehyde and eugenol on mononuclear cells of rheumatoid arthritis patients. Eur J Pharmacol. (2019) 852:14–24. doi: 10.1016/j.ejphar.2019.02.031, PMID: 30796902

[22] Abeysekera WPKM Arachchige SPG Abeysekera WKSM Ratnasooriya WD Medawatta HMUI. Antioxidant and glycemic regulatory properties potential of different maturity stages of leaf of Ceylon cinnamon (Cinnamomum zeylanicum blume) in vitro. Evid Complement Alternat Med. (2019) 2019:2693795. doi: 10.1155/2019/2693795 PMC6668558 31396287

[23] Kao TH Huang CW Chen BH. Functional components in Luffa cylindrica and their effects on anti-inflammation of macrophage cells. Food Chem. (2012) 135:386–95. doi: 10.1016/j.foodchem.2012.04.128, PMID: 22868104

[24] Waty S Suryanto D. Antibacterial activity of cinnamon ethanol extract (Cinnamomum burmannii) and its application as a mouthwash to inhibit streptococcus growth. IOP Conf Series: Earth Environ Sci. (2018) 130:012049. doi: 10.1088/1755-1315/130/1/012049

[25] SAS. SAS procedures and SAS/graph user’s guide, version 6. NC, USA: Statistical Analysis System Institute Inc., Cary. (2019).

[26] Abeysekera WPKM Premakumara GAS Ratnasooriya WD. In vitro antioxidant properties of leaf and bark extracts of Ceylon cinnamon (Cinnamomum zeylanicum blume). Trop Agric Res. (2013) 24:128–38.

[27] Rakasivi KGJ Chin KB. Antioxidant activity of Cinnamomum cassia extract and quality of raw chicken patties added with C. cassia powder and Pleurotus sajor-caju powder as functional ingredients during storage. Animal Biosci. (2022) 35:1279. doi: 10.5713/ab.21.0444, PMID: 35240027 PMC9262719

[28] Kim MJ Kang SE Jeong CH Min SG Hong SW Roh SW. Growth inhibitory effect of garlic powder and cinnamon extract on white colony-forming yeast in kimchi. Foods. (2021) 10:645. doi: 10.3390/foods10030645, PMID: 33803795 PMC8003234

[29] Dvorackova E Snoblova M Chromcova L Hrdlicka P. Effects of extraction methods on the phenolic compounds contents and antioxidant capacities of cinnamon extracts. Food Sci Biotechnol. (2015) 24:1201–7. doi: 10.1007/s10068-015-0154-4, PMID: 36080403

[30] Ho YS Wu JY Chang CY. A new natural antioxidant biomaterial from Cinnamomum osmophloeum Kaneshiro leaves represses melanogenesis and protects against DNA damage. Antioxidants. (2019) 8:474. doi: 10.3390/antiox8100474, PMID: 31614515 PMC6826928

[31] Shahpar B Moein M Zarshenas MM. Chemical assessment of eleven cinnamon aromatic water populations from Fars (Iran) local markets in comparison to a standard sample. Tr Pharm Sci. (2016) 2:131–8.

[32] Wang S Yang C Liao J Zhen W Chu F Chang S. Essential oil from leaves of Cinnamomum osmophloeum acts as a xanthine oxidase inhibitor and reduces the serum uric acid levels in oxonate-induced mice. Phytomedicine. (2008) 15:940–5. doi: 10.1016/j.phymed.2008.06.002, PMID: 18693097

[33] Lakshmi P Kumar GA. Nanosuspension technology: a review. Int J Pharm Pham Sci. (2010) 2:35–40.

[34] Clogston JD Patri AK. Zeta potential measurement. In: Characterization of nanoparticles intended for drug delivery – methods in molecular biology - Springer Protocols. Ed. McNeil S. E., Totowa, New Jersey, USA: Humana Press. (2011), 63–70 10.1007/978-1-60327-198-1_6 21116954

[35] Mukerjee A Pandey H Tripathi AK Singh SK. Development, characterization and evaluation of cinnamon oil and usnic acid blended nanoemulsion to attenuate skin carcinogenicity in swiss albino mice. Biocatal Agric Biotechnol. (2019) 20:101227. doi: 10.1016/j.bcab.2019.101227

[36] Ghosh V Saranya S Mukherjee A Chandrasekaran N. Cinnamon oil nanoemulsion formulation by ultrasonic emulsification: investigation of its bactericidal activity. J Nanosci Nanotechnol. (2013) 13:114–22. doi: 10.1166/jnn.2013.6701, PMID: 23646705

[37] Zhang S Zhang M Fang Z Liu Y. Preparation and characterization of blended cloves/cinnamon essential oil nanoemulsions. LWT-Food Sci Technol. (2017) 75:316–22. doi: 10.1016/j.lwt.2016.08.046

[38] Dávila-Rodríguez M López-Malo A Palou E Ramírez-Corona N Jiménez-Munguía MT. Antimicrobial activity of nanoemulsions of cinnamon, rosemary, and oregano essential oils on fresh celery. LWT-Food Sci Technol. (2019) 112:108247. doi: 10.1016/j.lwt.2019.06.014

[39] Tian WL Lei LL Zhang Q Li Y. Physical stability and antimicrobial activity of encapsulated cinnamaldehyde by self-emulsifying nanoemulsion. J Food Process Eng. (2016) 39:462–71. doi: 10.1111/jfpe.12237

[40] Quina FH Hinze WL. Surfactant-mediated cloud point extractions: an environmentally benign alternative separation approach. Ind Eng Chem Res. (1999) 38:4150–68. doi: 10.1021/ie980389n

[41] Zeng XS Geng WS Jia JJ. Neurotoxin-induced animal models of Parkinson disease: pathogenic mechanism and assessment. ASN Neuro. (2018) 10:1759091418777438. doi: 10.1177/1759091418777438, PMID: 29809058 PMC5977437

[42] Khasnavis S Pahan K. Cinnamon treatment upregulates neuroprotective proteins Parkin and DJ-1 and protects dopaminergic neurons in a mouse model of PD's disease. J Neuroimmune Pharmacol. (2014) 9:569–81. doi: 10.1007/s11481-014-9552-2, PMID: 24946862 PMC4167597

[43] Haleagrahara N Siew CJ Mitra NK Kumari M. Neuroprotective effect of bioflavonoid quercetin in 6-hydroxydopamine-induced oxidative stress biomarkers in the rat striatum. Neurosci Lett. (2011) 500:139–43. doi: 10.1016/j.neulet.2011.06.021, PMID: 21704673

[44] Karuppagounder SS Madathil SK Pandey M Haobam R Rajamma U Mohanakumar KP. Quercetin up-regulates mitochondrial complex-i activity to protect against programmed cell death in rotenone model of PD’s disease in rats. Neuroscience. (2013) 236:136–48. doi: 10.1016/j.neuroscience.2013.01.032, PMID: 23357119

[45] Ablat N Lv D Ren R Xiaokaiti Y Ma X Zhao X. Neuroprotective effects of a standardized flavonoid extract from safflower against a rotenone-induced rat model of PD's disease. Molecules. (2016) 21:1107. doi: 10.3390/molecules21091107, PMID: 27563865 PMC6274364

[46] Kumar R Kumar R Khurana N Singh SK Khurana S Verma S. Improved neuroprotective activity of fisetin through SNEDDS in ameliorating the behavioral alterations produced in rotenone-induced PD's model. Environ Sci Pollut Res Int. (2022) 29:50488–99. doi: 10.1007/s11356-022-19428-z, PMID: 35230633

[47] Dawson K. Nanoparticles in contact with living matter. International Workshop on Nanomedicines. London, United Kingdom, (2010)

[48] Tang J Xiong L Wang S Wang J Liu L Li J. Influence of silver nanoparticles on neurons and blood-brain barrier via subcutaneous injection in rats. Appl Surf Sci. (2008) 255:502–4. doi: 10.1016/j.apsusc.2008.06.058

[49] Betzer O Shilo M Opochinsky R Barnoy E Motiei M Okun E. The effect of nanoparticle size on the ability to cross the blood–brain barrier: an in vivo study. Nanomedicine. (2017) 12:1533–46. doi: 10.2217/nnm-2017-0022, PMID: 28621578

[50] Ohta S Kikuchi E Ishijima A Azuma T Sakuma I Ito T. Investigating the optimum size of nanoparticles for their delivery into the brain assisted by focused ultrasound-induced blood-brain barrier opening. Sci Rep. (2020) 10:18220. doi: 10.1038/s41598-020-75253-9 33106562 PMC7588485

[51] Chen Y Liu L. Modern methods for delivery of drugs across the blood–brain barrier. Adv Drug Deliv Rev. (2012) 64:640–65. doi: 10.1016/j.addr.2011.11.010, PMID: 22154620

[52] Pardridge WM. Blood–brain barrier drug targeting: the future of brain drug development. Mol Interv. (2003) 3:90–105. doi: 10.1124/mi.3.2.90, PMID: 14993430

[53] Sessa M Balestrieri ML Ferrari G Servillo L Castaldo D D’Onofrio N. Bioavailability of encapsulated resveratrol into nanoemulsion-based delivery systems. Food Chem. (2014) 147:42–50. doi: 10.1016/j.foodchem.2013.09.088, PMID: 24206683

[54] Gelperina SE Khalansky AS Skidan IN Smirnova ZS Bobruskin AI Severin SE. Toxicological studies of doxorubicin bound to polysorbate 80-coated poly (butyl cyanoacrylate) nanoparticles in healthy rats and rats with intracranial glioblastoma. Toxicol Lett. (2002) 126:131–41. doi: 10.1016/S0378-4274(01)00456-8, PMID: 11751017

[55] Nagpal K Singh SK Mishra DN. Nanoparticle mediated brain targeted delivery of gallic acid: in vivo behavioral and biochemical studies for protection against scopolamine-induced amnesia. Drug Deliv. (2013) 20:112–9. doi: 10.3109/10717544.2013.779330, PMID: 23651033

[56] Wohlfart S Gelperina S Kreuter J. Transport of drugs across the blood-brain barrier by nanoparticles. J Control Release. (2012) 161:264–73. doi: 10.1016/j.jconrel.2011.08.017, PMID: 21872624

[57] Raha S Dutta D Roy A Pahan K. Reduction of Lewy body pathology by oral cinnamon. J Neuroimmune Pharmacol. (2021) 16:592–608. doi: 10.1007/s11481-020-09955-2, PMID: 32889602 PMC7933354

[58] Saleem U Chauhdary Z Raza Z Shah S Rahman MU Zaib P. Anti-PD’s activity of Tribulus terrestris via modulation of ache, α-synuclein, TNF-α, and IL-1β. ACS Omega. (2020) 5:25216–27. doi: 10.1021/acsomega.0c03375, PMID: 33043200 PMC7542845

[59] Tikhonova MA Tikhonova NG Tenditnik MV Ovsyukova MV Akopyan AA Dubrovina NI. Effects of grape polyphenols on the life span and neuroinflammatory alterations related to neurodegenerative PD-like disturbances in mice. Molecules. (2020) 25:5339. doi: 10.3390/molecules25225339, PMID: 33207644 PMC7696792

[60] Guo YJ Dong SY Cui XX Feng Y Liu T Yin M. Resveratrol alleviates MPTP-induced motor impairments and pathological changes by autophagic degradation of α-synuclein via SIRT1-deacetylated LC3. Mol Nutr Food Res. (2016) 60:2161–75. doi: 10.1002/mnfr.201600111, PMID: 27296520 PMC6089356

[61] Pyo JH Jeong YK Yeo S Lee JH Jeong MY Kim SH. Neuroprotective effect of trans-cinnamaldehyde on the 6-hydroxydopamine-induced dopaminergic injury. Biol Pharm Bull. (2013) 36:1928–35. doi: 10.1248/bpb.b13-00537, PMID: 24292051

[62] Mehraein F Zamani M Negahdar F Shojaee A. Cinnamaldehyde attenuates dopaminergic neuronal loss in substantia nigra and induces midbrain catalase activity in a mouse model of PD’s disease. J Basic Clin Pathophysiol. (2018) 6:9–16.

[63] Ling L Jiang Y Liu Y Li H Bari A Ullah R. Role of gold nanoparticle from Cinnamomum verum against 1-methyl-4-phenyl-1, 2, 3, 6-tetrahydropyridine (MPTP) induced mice model. J Photochem Photobiol B. (2019) 201:111657. doi: 10.1016/j.jphotobiol.2019.111657, PMID: 31706085

[64] Kabuto H Tada M Kohno M. Eugenol (2-methoxy-4-(2-propenyl) phenol) prevents 6-hydroxydopamine-induced dopamine depression and lipid peroxidation inductivity in mouse striatum. Biol Pharm Bull. (2007) 30:423–7. doi: 10.1248/bpb.30.423, PMID: 17329831

[65] Sharma S Rabbani SA Narang JK Hyder Pottoo F Ali J Kumar S. Role of rutin nanoemulsion in ameliorating oxidative stress: pharmacokinetic and pharmacodynamics studies. Chem Phys Lipids. (2020) 228:104890. doi: 10.1016/j.chemphyslip.2020.104890, PMID: 32032570

[66] Kumar R Kumar R Khurana N Singh SK Khurana S Verma S. Enhanced oral bioavailability and neuroprotective effect of fisetin through its SNEDDS against rotenone-induced PD’s disease rat model. Food Chem Toxicol. (2020) 144:111590. doi: 10.1016/j.fct.2020.111590, PMID: 32710995

[67] Ramires Júnior OV Alves BDS Barros PAB Rodrigues JL Ferreira SP Monteiro LKS. Nanoemulsion improves the neuroprotective effects of curcumin in an experimental model of PD’s disease. Neurotox Res. (2021) 39:787–99. doi: 10.1007/s12640-021-00362-w, PMID: 33860897

[68] Churihar R More SA Mishra PS Gupta D. Evaluation of the effect of cinnamaldehyde per se and its interaction with ondansetron on haloperidol induced catalepsy in albino mice. Eur J Mol Clin Med. (2022) 9:580–5.

[69] Mekkey SM Raghif ARA Alkafaji HAR Hadi NR. The anti-PD effects of Cyanara scoluymus (artichoke) extract in rat model of rotenone induced PDism. Ann Romanian Soc Cell Biol. (2021) 25:2318–29.

[70] Wadman M. FDA no longer has to require animal testing for new drugs. Science. (2023) 379:127–8. doi: 10.1126/science.adg6276, PMID: 36634170

[71] Banks WA. From blood-brain barrier to blood-brain interface: new opportunities for CNS drug delivery. Nat Rev Drug Discov. (2016) 15:275–92. doi: 10.1038/nrd.2015.21, PMID: 26794270

[72] Feigin VL Vos T Nichols MO Owolabi WM Carroll M Dichgans G. The global burden of neurological disorders: translating evidence into policy. Lancet Neurol. (2020) 19:255–65. doi: 10.1016/S1474-4422(19)30411-9, PMID: 31813850 PMC9945815

[73] Perez-Lopez A Torres-Suarez AI Martin-Sabroso C Aparicio-Blanco J. An overview of in vitro 3D models of the blood-brain barrier as a tool to predict the in vivo permeability of nanomedicines. Adv Drug Deliv Rev. (2023) 196:114816. doi: 10.1016/j.addr.2023.114816, PMID: 37003488

[74] Williams-Medina A Deblock M Janigro D. In vitro models of the blood-brain barrier: tools in translational medicine. Front Med Technol. (2021) 2:623950. doi: 10.3389/fmedt.2020.623950 35047899 PMC8757867

