# Supplementary figures and images for: Analysis of bioactive compounds in cinnamon leaves and preparation of nanoemulsion and byproducts for improving Parkinson’s disease in rats
Source: Front Nutr. 2023 Aug 2;10:1229192. doi: 10.3389/fnut.2023.1229192 (PMC10433916; doi:10.3389/fnut.2023.1229192)

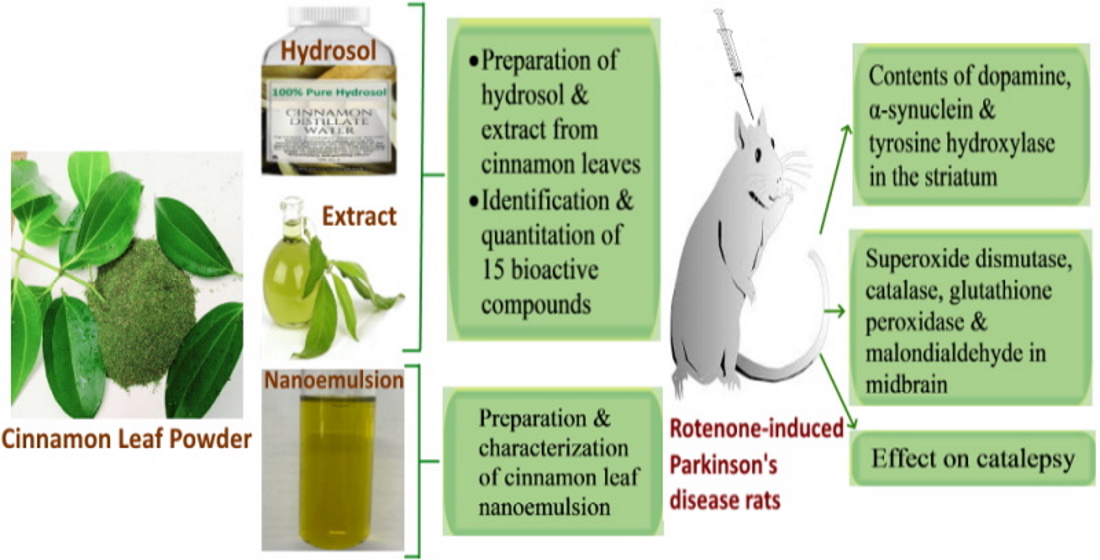

Supplement: Supplementary file 1 [file Image_1.JPEG]
